# Will Nigerians Win the War Against Urinary Schistosomiasis? Prevalence, Intensity, Risk Factors and Knowledge Assessment among Some Rural Communities in Southwestern Nigeria

**DOI:** 10.3390/pathogens9020128

**Published:** 2020-02-17

**Authors:** Oluwaseun B. Awosolu, Yahaya Z. Shariman, Farah Haziqah M. T., Titus A. Olusi

**Affiliations:** 1 School of Biological Sciences, Universiti Sains Malaysia, Penang 11800 USM, Malaysia; zary@usm.my (Y.Z.S.); farahhaziqah@usm.my (F.H.M.T.); 2 Parasitology and Public Health Unit, Department of Biology, Federal University of Technology, Akure PMB 704, Nigeria; taolusi@futa.edu.ng

**Keywords:** urinary schistosomiasis, *Schistosoma haematobium*, rural communities, prevalence, intensity, risk factors, knowledge assessment, Nigeria

## Abstract

Urinary schistosomiasis is a devastating parasitic disease in Nigeria. This study was carried out to investigate the current prevalence, intensity, risk factors and knowledge assessment among some rural communities in southwestern Nigeria. A cross-sectional study was carried out in which a standard urine filtrations technique was used to determine the prevalence and intensity of infection. A well-designed questionnaire was used to collect subject’s data on demographic and socioeconomic characteristics. Of the total 620 urine samples examined, overall 346 (55.81%) were positive with a mean egg intensity (S.D) of 65.60 (59.33) egg/10 mL of urine. Significant differences occurred in the analysis. Males had the highest prevalence and intensity of 224 (61.9%) and 69.20 egg/10 mL of urine, respectively. The 10–14 years age group had the highest prevalence of 65.9% while mean intensity of infection among the age group decreases with increasing age, with the highest mean intensity of infection (80.14 egg/10 mL) recorded among the age group ≤ 4 years. Bivariate logistic regression analysis showed that being age group 10–14 (COR 0.27, 95% CI: 0.09–0.79) and dependent on river (COR 0.67, 95% CI: 0.33–1.33) increased the odd of contracting an infection. Similarly, the knowledge of respondents on urinary schistosomiasis was low. Conclusively, urinary schistosomiasis is still persistent at a very high rate in the study area and appropriate control measure should be deployed.

## 1. Introduction 

Schistosomiasis, also known as bilharziasis, is a waterborne parasitic disease caused by parasites of the genus *Schistosoma* and it is a public health concern worldwide, particularly in Sub-Saharan Africa. The major schistosome species include *Schistosoma haematobium*, which is the most widespread in Sub-Saharan Africa [1,2,3,4] and responsible for urinary schistosomiasis, while *Schistosoma mansoni, S. intercalatum, S. japonicum* and *S. mekongi* are all responsible for intestinal schistosomiasis [5]. Schistosomiasis is transmitted by some specific types of freshwater snail such as *Bulinus* spp. and *Biophalaria* spp. [5,6,7]. An adult, well-developed *S. haematobium* is cylindrical and elongated in shape and it is made up of a male and a female paired together to form a copula. The slender female is carried in the male gynaecophoric canal and it is highly pathogenic. This is because the egg with a terminal spine can induce morbidity and cause a granulomatous lesions due to the host immune response. Thus, the WHO International Agency for Research on Cancer (IARC) has declared *S. haematobium* as a group 1 carcinogen [7,8,9]. The lifecycle starts when an egg hatches into a free-living miracidium on reaching fresh water under favourable conditions. The miracidium then locates and infect a suitable snail host (*Bulinus* spp.) inside where it undergoes asexual development to form mother and daughter sporocysts which eventually form cercariae and are released into water bodies. The cercariae infect a suitable mammalian host such as man by penetrating through the skin. On getting into the host, it migrates to the liver and eventually into the venous plexus of the bladder where the female produces its eggs [7]. The transmission of urinary schistosomiasis in Nigeria and all over the world is determined by some risk factors such as socioeconomic, environmental, climatic, behavioural, and cultural factors [10,11,12,13,14]. 

Schistosomiasis is an insidious but highly devastating parasitic disease that affects over 250 million people throughout the world with an estimated global burden of 1.4 million disability-adjusted life years (DALYs) in year 2017 [15,16,17]. Moreover, it is estimated that over 200,000 people die as a result of the disease every year. The Sub-Saharan region of Africa alone accounts for an estimated 90% of total worldwide infections [4,7,18]. In Nigeria, the disease is prevalent throughout the country, with an estimated 25 million people infected and 101 million at risk of infection, respectively [19]. Furthermore, about 30 million people have been deemed to be in dire need of treatment annually. *S. haematobium* infection in Nigeria varies between 2 to 90%, of which most cases occur among school-going children and those with very low socioeconomic status [12,20]. Although, in Osun and Kwara State, urinary schistosomiasis has been studied and reported to some extent in several rural and peri-urban communities [21,22,23,24,25], however, these studies are not sufficient to determine the factors affecting the distribution of urinary schistosomiasis in these areas. Moreover, there is an urgent need for updated epidemiological information that can enhance the management, control and prevention of urinary schistosomiasis in Osun and Kwara State. To fill this gap, this current study focuses on the prevalence, intensity, risk factors and knowledge assessment affecting the distribution of urinary schistosomiasis among some selected rural communities in Southwestern Nigeria.

## 2. Results 

### 2.1. Demographic Characteristics of Participants

Out of the 652 individuals approached, 620 submitted urine samples and were enrolled in the study. There were considerably more males than females (58.4% vs. 41.6%) which make the sex ratio of participants to be 1.4. The ages of participants range between 3 and 22 years (mean ± SD = 12.1 ± 3.8) and median age was 13years while age group 10–14 years had more subjects than others (43.5%). it was obvious that most of the participants (55.5%) depend on rivers/streams for daily activities such as washing (Table 1). 

### 2.2. Prevalence and Intensity of Urinary Schistosomiasis in Relation to Sex and Age among the Study Population

Out of the 620 subjects examined for urinary schistosomiasis, overall 346 (55.81%) were observed to be infected with a mean egg intensity (S.D) of 65.60 (59.33) egg/10 mL of urine. Males had the highest prevalence and mean intensity (S.D) of 224 (61.9%) and 69.29 (68.9) egg/10 mL of urine respectively. Additionally, it was observed that over half (53.24%) of those infected had a history of previous infection (Table 2).

Among the age groups, the highest prevalence of infection was recorded among the 10–14 year age group, with a prevalence of 178 (65.9%), while participants aged 20 years and above had the least (33.3%) prevalence of *S. haematobium* infection. Moreover, the mean intensity of infection among the various age group gradually decrease with increasing age with the highest mean intensity of 80.14 egg/10 mL of urine among age group ≤ 4 years and the least among age group ≥ 20 years (Table 2). The differences were statistically significant (*p* < 0.05). 

### 2.3. Prevalence and Intensity of Urinary Schistosomiasis in Relation to Study Communities

**Figure 1:**
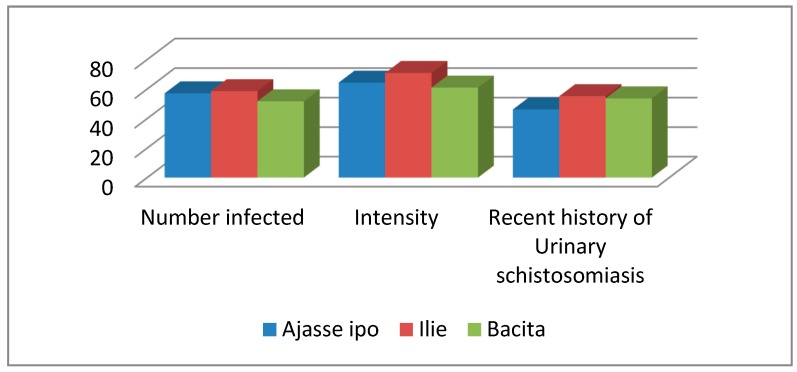
Prevalence, intensity and recent history of *Schistosoma haematobium* infection among sampled communities.

The prevalence of infection in the three study communities is shown in Figure 1. The highest prevalence of 58.6% and the highest mean intensity of 70.91 egg/10 mL of urine were recorded in Ilie community with 55.2% of the subjects having previous infection with *S. haematobium* though there was no significant difference (χ2 = 2.187; *p* > 0.05).

### 2.4. Prevalence and Intensity of Urinary Schistosomiasis in Relation to Socioeconomic Factors Affecting the Transmission of Urinary Schistosomiasis among the Study Population

The prevalence and intensity of urinary schistosomiasis in relation to socioeconomic factors affecting urinary schistosomiasis is presented in Table 3. It was observed that those whose father’s occupation is farming are more exposed to the disease with a prevalence and mean intensity of 66.2% and 64.71 egg/10 mL of urine. Moreover, those whose mothers are farmers also had higher prevalence (76.8%) and mean intensity of infection (75.67 egg/10 mL). Significant differences occurred in the analysis (*p* < 0.05).

In the same vein, those whose parents are uneducated are more likely to have urinary schistosomiasis. Main source of water supply and water contact activities have great impact on the transmission of urinary schistosomiasis. Obviously, those who depend majorly on river or stream water and still get involved in agricultural work tend to harbor more infection than others. 

Bivariate logistic regression analysis of factors associated with urinary schistosomiasis among the study population shows that age, main source of water supply and water contact activities are closely associated with urinary schistosomiasis (Table 4).

### 2.5. Knowledge of Respondents on Urinary Schistosomiasis among the Study Population

The knowledge of respondents on urinary schistosomiasis in the study area is shown in Table 5. Generally, only some of the respondents are well informed about *S. haematobium* infection. It was recorded that in spite the fact that almost one-fifth of respondents (19.4%) knew that the source of infection is through contact with contaminated rivers or streams, others did not know the source of infection and the intermediate host of *S. haematobium*. Similarly, while many respondents (34.4%) are aware of the symptoms of infection to be blood in the urine, a considerable number of the respondents (87.1%) do not know preventive measures against urinary schistosomiasis. (see Supplementary Materials).

## 3. Discussion 

In Nigeria, urinary schistosomiasis is a major public health issue which has been confirmed in different parts of the country and the prevalence is on the increase on a daily basis. The present study is not an exception as our study reveals and corroborates previous reports on the endemicity of infection in the study area. The prevalence of urinary schistosomiasis recorded in this study is 55.81% which falls within the national Nigerian prevalence range of 2 to 82.5% but higher than the national mean of 13% in Nigeria [26,27]. This is consistent with previous studies conducted in Nigeria and other countries in tropical regions [12,14,23,28,29]. On the other hand, it is higher than the reports from many other studies [30,31,32,33,34,35]. This could be attributed to types of water contact activities, the socioeconomic status of the subjects and the different ecological settings in the different geographical areas. Worthy of note is that over half (53.24%) of those infected had a history of previous infection and treatment which means that reinfection is a stable pattern and a cause for concern in the study area. Obviously, there was prior individual case treatment in which some of the participants were given praziquantel (40 mg/kg) for treatment at community health center about three years before this study. However, due to repeated exposure to contaminated open streams or rivers without consistent treatment, there was reinfection in the study area. The mean intensity of 65.60 egg/10 mL of urine indicates that infection is long-standing, deep-rooted and well established. This could be due to subjects’ constant exposure to contaminated water bodies without any chemotherapeutic treatment and preventive measures. *S. haematobium* infection intensity reports have shown that majority of infected persons have light to moderate infection which is similar to this study [36].

Our findings revealed that the prevalence and mean intensity of infection was higher among male subjects than females and this is similar to previous reports [37,38,39]. On the other hand, other studies have reported that females have higher infection rates than males [40,41]. The higher infection among males than female in this present study is probably a result of gender-specific water-contact activities in which males tend to get more involved such as fishing, swimming and playing in water naked unlike their female counterparts, thereby exposing them to higher infection rates than their females.

The age pattern of prevalence of infection shows that 10–14 years age group had the highest prevalence and intensity of 65.9% and 67.37 egg/10 mL of urine, respectively. This is because members of this age group tend to joyfully engage in several water contact activities such as swimming, bathing, fishing and playing in schistosome-infested open water bodies that enhance their exposure to infection [14,15]. Additionally, the mean intensity of infection decreases with increasing age. Thus the highest mean intensity of 80.14 egg/10 mL of urine was recorded among age group ≤ 4. This is because this age group has little or no immunity compared to older ones that have developed partial immunity as a result of long-term exposure to infections [42].

Among the study communities, Ilie which is a community situated close to a dam named ‘Erinle’ had the highest prevalence and mean intensity of infection. This is in tandem with other previous studies [2,12,42,43]. It is obvious from our study that communities situated beside dams usually have the tendency to be more exposed to urinary schistosomiasis because of changes in ecological settings that lead to increase in intermediate snail host which harbor infection and enhance transmission. Moreover, such communities tend to actively engage in water contact activities [2,44,45,46].

The prevalence of urinary schistosomiasis in the study area vary significantly to father’s occupation, mother’s occupation, father’s education, mother’s education, main source of water supply and water-contact activities. Those whose fathers are farmers harboured the highest prevalence of infection, but mean intensity was highest among those whose fathers engage in fishing as an occupation. Children, especially male tend to join their father in their occupational activities. Thus, when children join their father in farming and fishing activities, they are exposed to infection. Just as father’s occupation have great impact on transmission of urinary schistosomiasis, so also mother’s occupation. Thus, parent’s occupation plays major role in transmission of infection and this is in agreement with previous studies [34]. Furthermore, parent’s education had great effect on the prevalence of infection in our study since the highest level of infection occurred among those whose parents did not complete primary school education compared to others. This agrees with the reports of [47] and [48]. Similarly, we cannot overemphasize the impact of source of water supply in which over 74.3% of those who depend on river or stream had infection with a high mean intensity of infection. In order to control urinary schistosomiasis, appropriate source of water supply for domestic use must be provided at a reasonable distance. This may prevent most of the community members from having close contact with contaminated rivers or streams. Those who engage in agricultural work and fishing as a major water-contact activity are more exposed to infection than others. Preventive measures should be provided for those who cannot avoid exposure to infection due to the nature of their work. Finally, our study showed that age, main source of water and water-contact activities are major risk factors associated with contracting *S. haematobium* infection as reported previously by [12]. Generally, knowledge of respondents on urinary schistosomiasis in the study area is low and it is a cause for concern. There is a popular saying that “if one is not informed, one is deformed”. Thus, to avoid uninterrupted transmission of infection, appropriate information through proper education on urinary schistosomiasis should constantly be disseminated through the media. Additionally, functional tap water should be provided as this may reduce the rate at which residents are visiting contaminated streams or river while infected individuals should be consistently treated with praziquantel (40 mg/kg) at least once a year. 

## 4. Materials and Methods

### 4.1. Study Area

**Figure 2:**
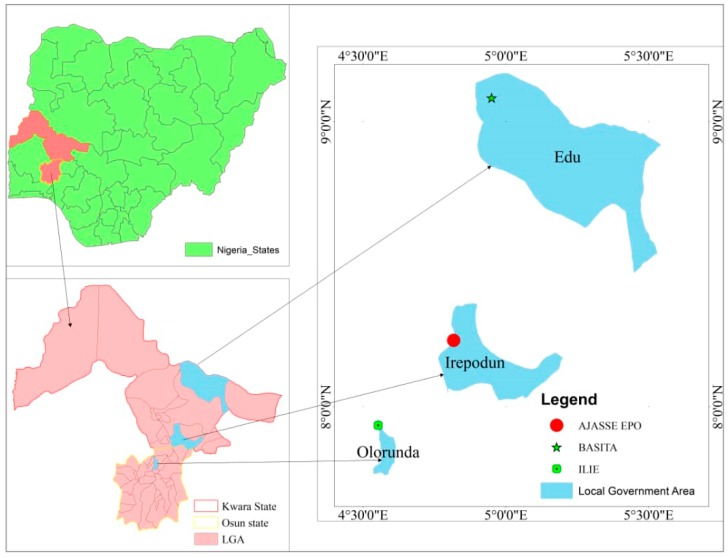
Map of Nigeria indicating the study area.

The study was conducted in three selected rural communities in Southwest Nigeria which include Ilie, Ajasse ipo and Bacita. Ilie is a rural community located in the rain forest zone of Osun State which connects via Ajasse Ipo-Osogbo road. It is situated between Latitude 4°34′ and 4°36′ E, and Longitude 7°56′ and 7°58′ N and has a population of approximately 2268. It is about 27.6 km from Osogbo, Osun State capital. Similarly, Ajasse ipo is located between Latitude 8°13′ 60 N and Longitude 4°49′ 0 E and serves as a major link between Kwara State and others such as Ekiti and Osun State. It is approximately 43.8 km from Ilorin, the State capital. The population is estimated to be 8,953. On the other hand, Bacita is situated between Latitude 9°4′ 59.99″ N and Longitude 4°57′ 0.00″ E (Figure 2) and the population is approximately 2541 [49]. The distance is estimated to be 78 km from Ilorin. These communities are all in tropical region with a local climate of dry season (November-March) and rainy season (April-October). Each of this communities has major river upon which the local residents depends for their source of daily water supply and other activities such fishing and farming. The communities lack basic amenities and, the health care centers are rarely accessible since it is far and roads are in a deplorable condition.

### 4.2. Study Population, Design and Sample Size

A community-based cross-sectional study was conducted in which each member of the house was selected at random. The minimum population size was 384 considering the total population of the communities and a 5% margin of error based on the recommendation of [50]. However, 620 samples were collected to minimize error.

### 4.3. Urine Collection and Examination for Schistosoma haematobium Egg

Urine sample collection was carried out at 10:00 and 14:00 hours of the day, when the body’s metabolic activity is relatively high. Samples were obtained from each volunteer individuals in a well-labelled, sterilized, wide-mouthed, tight-capped plastic container. Samples were transported to the laboratory immediately after collection for analysis. In the laboratory, urine specimens were processed by the standard filtration technique in which 10 mL of each sample was passed through a 12-μm pore filter membrane so as to retain the *S. haematobium* eggs contained, which is thereafter examined, viewed and counted under a 40x objective lens of a light microscope to identify the ova which are characterized by a terminal spine. The mean egg intensity of infection was categorized as heavy (≥ 500 egg/10 mL), moderate (51 to 499 egg/10 mL) and light (≤ 50 eggs/10 mL) in line with the current World Health Organization classification [51]. 

### 4.4. Examination of Urine for Microhaematuria and Proteinuria

Microhaematuria and proteinuria were examined by gently dipping a commercially available reagent strip (Medi-test combur-9; Analyticon Biotecnologies, Lichtenfels, Germany) into the urine sample contained in sterile plastic bottle for 5 s in accordance with the manufacturer’s instructions. The colour change was then compared with the manufacturer’s colour chart to estimate the amount of blood in urine.

### 4.5. Focus Group Discussions (FGDs) and Questionnaire Administration

Focus group discussions (FGDs) were held with parents, caregivers or guardians, primary health care officials and even the community heads prior the administration of questionnaires. The interview focused on perceptions on urinary schistosomiasis, knowledge, attitude, practices and water contact activities in the area. During questionnaire administration, community members were grouped into five based on their age which includes ≤ 4 years, 5–9 years, 10–14 years, 15–19 years and ≥ 20 years. Each parent or guardians of children aged ≤ 4 years and 5–9 years were interviewed using the local language after which questionnaires for each child was filled. Individuals aged 10 years and above were given questionnaires to fill under the guide of trained personnel. Information on sociodemographic factors such as sex, age, father’s occupation, mother’s occupation, educational status and water contact activities such as swimming, fishing, playing or bathing, fetching water for domestic use, etc., were collected through a well-designed questionnaire.

### 4.6. Statistical Analysis

The Statistical Package for Social Sciences (SPSS) for Windows software package version 16.0 (SPSS Inc, Chicago, IL, USA) was used to perform the data analysis. Comparisons of prevalence by age and sex were done using chi square tests. The mean egg count was explored using Student’s t-test and one-way ANOVA. *p*-values < 0.05 were considered statistically significant. The analysis of the relationship between prevalence of urinary schistosomiasis and risk factors in order to determine the crude odds ratio (ORs) and 95% confidence interval were explored using logistic regression analysis.

### 4.7. Ethical Clearance

Prior to the commencement of the study, the Research and Ethical Committee of the University of Ilorin Teaching Hospital, ad-hoc ethical committee of the Local Government Authorities (LGA) and the traditional leaders of each community reviewed and approved the study protocol. Just before the study commences, individual participants including the guardian or caregiver of each child and the village heads were fully educated on the objective, procedures, benefits and risks of the study. immediately after this, written informed consent was obtained from each adult subject and the guardians or caregivers of each child investigated before their enrollment in the study.

## 5. Conclusions

Urinary schistosomiasis is a serious endemic disease in this study area and should not be handled with levity. Major risk factors contributing to infection include poor knowledge of urinary schistosomiasis, water contact activities such as washing, occupation such as farming and fishing and, lack of functional tap water. As such appropriate management control measures and intervention such as functional pipe borne water, increased health education on urinary schistosomiasis, adequate treatment with praziquantel and improved health care centers should be made available to these rural communities. All these will go a long way to alleviate this devastating plague in the study area.

## Figures and Tables

**Table 1:** Socio-demographic characteristics of the study participants (n = 620).

Variables	N	%
**Gender**		
Male	362	58.4
Female	258	41.6
**Age**		
≤4	17	2.7
9–5	156	25.2
14–10	270	43.5
15–19	168	27.1
≥20	9	1.5
**Father’s Occupation**		
Fishing	15	2.4
Farming	263	42.4
Trading	161	26
Salary earner	132	21.3
Wage earner	49	7.9
**Mother’s Occupation**		
Trading	291	46.9
Farming	138	22.3
Salary earner	129	20.8
Unemployed	62	10
**Does Your Father Complete Primary Education**		
Yes	477	76.9
No	143	23.1
**Does Your Mother Complete Primary Education**		
Yes	432	69.7
No	188	30.3
**Main Source of Water Supply**		
Tap	71	11.5
Well	309	49.8
River	237	38.2
Others	3	0.5
**Water Contact Activities**		
Playing/Bathing	137	22.1
Washing	344	55.5
Agricultural work	51	8.2
Fishing	13	2.1
No contact	75	12.1
**Community**		
Ajasse Ipo	212	34.2
Ilie	203	32.7
Bacita	205	33.1
**TOTAL**	**620**	**100**

**Table 2:** Prevalence and intensity of urinary schistosomiasis in relation to sex and age among the study population.

Variables	No Examined N	No Infected n (%)	Mean (S.D) Intensity of Infection	Recent History of Urinary Schistosomiasis n (%)
**Sex**				
Male	362	224 (61.9)	69.29 (68.9)	193 (53.3)
Female	258	122 (47.3)	58.81 (34.6)	127 (49.2)
χ2		13.005		1.009
*p* value		< 0.001	0.116	0.315
**Age (Years)**				
≤4	17	7 (41.2)	80.14 (54.2)	8 (47.1)
5–9	156	84 (53.8)	77.77 (97.2)	80 (51.3)
10–14	270	178 (65.9)	67.37 (44.1)	159 (58.9)
15–19	168	74 (44.0)	47.43 (18.6)	71 (42.3)
≥20	9	3 (33.3)	33.67 (11.8)	2 (22.2)
χ2		24.191		14.867
*p* value		< 0.001	0.017	*p* = 0.005
**Total**	**620**	**346 (55.81)**	**65.60 (59.33)**	**320 (53.24)**

S.D = Standard Deviation.

**Table 3:** Prevalence and intensity of urinary schistosomiasis in relation to socioeconomic factors among the study population.

Variables	No Examined	No Infected (%)	Mean (S.D) Intensity of Infection	Recent History of Urinary Schistosomiasis (%)
**Father’s Occupation**				
Fishing	15	9 (60.0)	109.33 (110.68)	9 (60.0)
Farming	263	174 (66.2)	64.71 (63.46)	136 (51.7)
Trading	161	86 (53.4)	61.43 (41.27)	80 (49.7)
Salary earner	132	55 (41.7)	68.76 (64.42)	70 (53.0)
Wage earner	49	22 (44.9)	63.09 (38.55)	25 (51.0)
χ2		24.975		0.775
*p* value		< 0.001	0.236	*p* = 0.942
**Mother’s Occupation**				
Trading	291	156 (53.6)	59.33 (38.35)	144 (49.5)
Farming	138	106 (76.8)	75.67 (81.83)	77 (55.8)
Salary earner	129	50 (38.8)	65.00 (66.97)	59 (45.7)
Unemployed	62	34 (54.8)	63.82 (37.56)	40 (64.5)
χ2		40.481		7.412
*p* value		< 0.001	0.185	*p* = 0.060
**Does Your Father Complete Primary Education**				
Yes	477	242 (50.7)	58.87 (41.47)	234 (49.1)
No	143	104 (72.7)	81.26 (86.09)	86 (60.1)
χ2		21.578		5.411
*p* value		< 0.001	< 0.001	0.02
**Does Your Mother Complete Primary Education**				
Yes	432	214 (49.5)	59.88 (43.83)	209 (48.4)
No	188	132 (70.2)	74.86 (77.50)	111 (59.0)
χ2		22.705		5.964
*p* value		< 0.001	= 0.022	*p* = 0.015
**Main Source of Water Supply**				
Tap	71	35 (49.3)	53.37 (24.01)	38 (53.5)
Well	309	134 (43.4)	63.03 (50.92)	144 (46.6)
River/stream	237	176 (74.3)	70.07 (69.28)	137 (57.8)
Others	3	1 (33.3)	51	1 (33.3)
χ2		53.956		7.251
*p* value		< 0.001	0.425	0.064
**Water Contact Activities**				
Playing/Bathing	137	84 (61.3)	67.69 (63.17)	62 (45.3)
Washing	344	214 (62.2)	60.70 (47.28)	187 (54.4)
Agricultural work	51	39 (76.5)	82.82 (93.08)	29 (56.9)
Fishing	13	8 (61.5)	92.00 (88.63)	7 (53.8)
No contact	75	1 (1.3)	56	35 (46.7)
χ2		1.066		4.581
*p* value		< 0.001	0.167	0.333
**TOTAL**	**620**	**346 (55.8)**	**65.60 (59.33)**	**320 (53.24)**

**Table 4:** Bivariate logistic regression analysis of factors associated with urinary schistosomiasis among the study population.

Variables	OR (95% CI)	*p*-value
**Gender**		
Male	1	
Female	1.59 (1.07–2.36)	0.022
**Age**		
≤4	1	0
5–9	0.30 (0.10–0.93)	0.038
10–14	0.27 (0.09–0.79)	0.017
15–19	0.80 (0.27–2.39)	0.693
≥20	1.52 (0.24–9.65)	0.656
**Father’s Occupation**		
Fishing	1	0.874
Farming	0.90 (0.23–3.46)	0.879
Trading	0.72 (0.17–2.89)	0.643
Salary earner	0.69 (0.16–2.97)	0.625
Wage earner	0.91 (0.20–4.13)	0.905
**Mother’s Occupation**		
Trading	1	0.266
Farming	0.56 (0.29–1.07)	0.08
Salary earner	1.10 (0.61–1.96)	0.748
Unemployed	0.69 (0.33–1.41)	0.313
**Does Your Father Complete Primary Education**		
No		
**Does Your Mother Complete Primary Education**	0.88 (0.46–1.65)	0.69
No		
**Main Source of Water Supply**		
Tap	0.96 (0.54–1.70)	0.897
Well		
River	1	0.003
Others	1.53 (0.80–2.92)	0.189
**Water Contact Activities**	0.67 (0.33–1.33)	0.259
Playing/Bathing	0.74 (0.04–13.24)	0.841
Washing		
Agricultural work	1	0
Fishing	0.96 (0.61–1.51)	0.871
	0.69 (0.30–1.57)	0.386
	1.00 (0.24–4.15)	0.992

OR = Odd Ratio, CI = Confidence Interval.

**Table 5:** Knowledge of respondents about urinary schistosomiasis in the study area.

Variables	Number Examined	(%)
**Route of Schistosome Infection**		
Contact with contaminated natural water	128	20.6
Eating unhygienic food	40	6.5
Playing with soil	45	7.3
I don’t know	407	65.6
**Source of Infection**		
River water	120	19.4
Playing with infected friends	62	10
Foods	53	8.5
I don’t know	385	62.1
**Intermediate Host**		
House fly	282	45.5
Water snail	99	16
Fish	135	21.8
House rat	51	8.2
I don’t know	53	8.5
**Symptoms of Infection**		
Blood in urine	213	34.4
Stomach pain	231	37.3
Waist pain	89	14.4
Blurred vision	27	4.4
I don’t know	60	9.7
**Prevention of Infection**		
Stop going to river water	80	12.9
Eating good food	188	30.3
Bathing regularly	63	10.2
Treating drinking water	206	33.2
I don’t know	83	13.4
**Total**	620	

## Supplementary Materials

The following are available online at https://www.mdpi.com/2076-0817/9/2/128/s1, Figure S1: 1 Prevalence, intensity and recent history of S. haematobium infection among sampled communities, Table S1: Knowledge of urinary schistosomiasis with respect to age, sex, father’s occupation, mother’s occupation, father’s education, mother’s education, water contact activities and main source of water supply.
