# Supplementary material for: Will Nigerians Win the War Against Urinary Schistosomiasis? Prevalence, Intensity, Risk Factors and Knowledge Assessment among Some Rural Communities in Southwestern Nigeria
Source: Pathogens. 2020 Feb 17;9(2):128. doi: 10.3390/pathogens9020128 (PMC7168148; doi:10.3390/pathogens9020128)
Supplement: Supplementary file 1 [file pathogens-09-00128-s001.pdf]

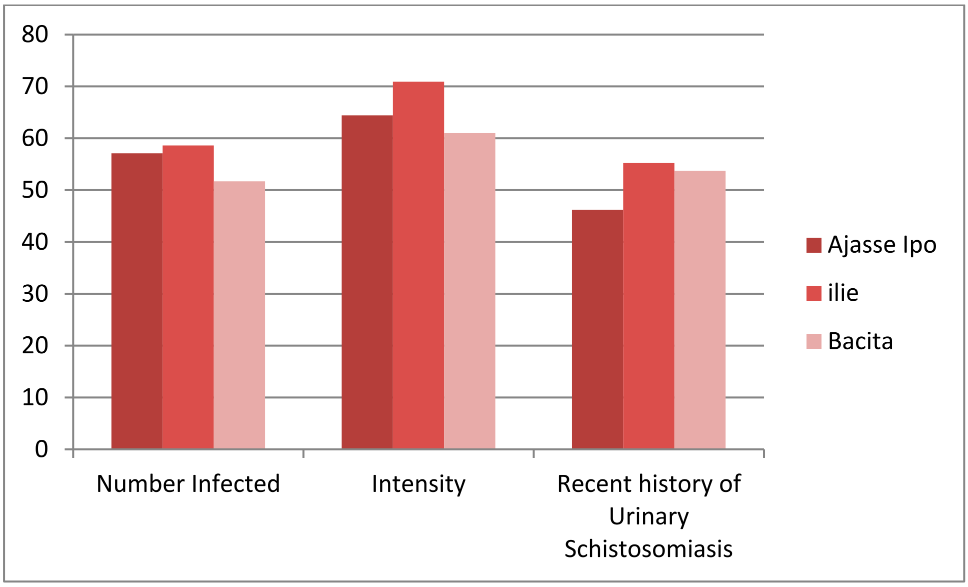

Figure S1. Prevalence, intensity and recent history of S. haematobium infection among sampled communities.

Table S1. Knowledge of urinary schistosomiasis with respect to age, sex, father's occupation, mother's occupation, father's education, mother's education, water contact activities and main source of water supply.

Crosstab

			main source of water supply				Total
			tap	well	river	others	
prevention of infection	stop going to river	Count	14	45	20	1	80
		Expected Count	9.2	39.9	30.6	.4	80.0
		% within prevention of infection	17.5%	56.2%	25.0%	1.2%	100.0%
		% within main source of water supply	19.7%	14.6%	8.4%	33.3%	12.9%
		% of Total	2.3%	7.3%	3.2%	.2%	12.9%
	eating good food	Count	18	101	69	0	188
		Expected Count	21.5	93.7	71.9	.9	188.0
		% within prevention of infection	9.6%	53.7%	36.7%	.0%	100.0%
		% within main source of water supply	25.4%	32.7%	29.1%	.0%	30.3%
		% of Total	2.9%	16.3%	11.1%	.0%	30.3%
	bathing regularly	Count	5	23	35	0	63
		Expected Count	7.2	31.4	24.1	.3	63.0
		% within prevention of infection	7.9%	36.5%	55.6%	.0%	100.0%
		% within main source of water supply	7.0%	7.4%	14.8%	.0%	10.2%
		% of Total	.8%	3.7%	5.6%	.0%	10.2%
	treat drinking water	Count	16	104	86	0	206
		Expected Count	23.6	102.7	78.7	1.0	206.0
		% within prevention of infection	7.8%	50.5%	41.7%	.0%	100.0%
		% within main source of water supply	22.5%	33.7%	36.3%	.0%	33.2%
		% of Total	2.6%	16.8%	13.9%	.0%	33.2%
	i don'know	Count	18	36	27	2	83

	Expected Count	9.5	41.4	31.7	.4	83.0
	% within prevention of infection	21.7%	43.4%	32.5%	2.4%	100.0%
	% within main source of water supply	25.4%	11.7%	11.4%	66.7%	13.4%
	% of Total	2.9%	5.8%	4.4%	.3%	13.4%
Total	Count	71	309	237	3	620
	Expected Count	71.0	309.0	237.0	3.0	620.0
	% within prevention of infection	11.5%	49.8%	38.2%	.5%	100.0%
	% within main source of water supply	100.0%	100.0%	100.0%	100.0%	100.0%
	% of Total	11.5%	49.8%	38.2%	.5%	100.0%

prevention of infection * main source of water supply

Crosstab

			contact made with water					Total
			play or bath	washing	agricultural work	fishing	no contact	
prevention of infection	stop going to river	Count	23	42	3	1	11	80
		Expected Count	17.7	44.4	6.6	1.7	9.7	80.0
		% within prevention of infection	28.8%	52.5%	3.8%	1.2%	13.8%	100.0%
		% within contact made with water	16.8%	12.2%	5.9%	7.7%	14.7%	12.9%
		% of Total	3.7%	6.8%	.5%	.2%	1.8%	12.9%
	eating good food	Count	31	104	19	7	27	188
		Expected Count	41.5	104.3	15.5	3.9	22.7	188.0
		% within prevention of infection	16.5%	55.3%	10.1%	3.7%	14.4%	100.0%
		% within contact made with water	22.6%	30.2%	37.3%	53.8%	36.0%	30.3%
		% of Total	5.0%	16.8%	3.1%	1.1%	4.4%	30.3%
	bathing regularly	Count	11	33	9	4	6	63
		Expected Count	13.9	35.0	5.2	1.3	7.6	63.0
		% within prevention of infection	17.5%	52.4%	14.3%	6.3%	9.5%	100.0%
		% within contact made with water	8.0%	9.6%	17.6%	30.8%	8.0%	10.2%
		% of Total	1.8%	5.3%	1.5%	.6%	1.0%	10.2%
		Count	44	125	13	1	23	206

	treat drinking water	Expected Count	45.5	114.3	16.9	4.3	24.9	206.0
		% within prevention of infection	21.4%	60.7%	6.3%	.5%	11.2%	100.0%
		% within contact made with water	32.1%	36.3%	25.5%	7.7%	30.7%	33.2%
		% of Total	7.1%	20.2%	2.1%	.2%	3.7%	33.2%
	i don'know	Count	28	40	7	0	8	83
		Expected Count	18.3	46.1	6.8	1.7	10.0	83.0
		% within prevention of infection	33.7%	48.2%	8.4%	.0%	9.6%	100.0%
		% within contact made with water	20.4%	11.6%	13.7%	.0%	10.7%	13.4%
		% of Total	4.5%	6.5%	1.1%	.0%	1.3%	13.4%
Total		Count	137	344	51	13	75	620
		Expected Count	137.0	344.0	51.0	13.0	75.0	620.0
		% within prevention of infection	22.1%	55.5%	8.2%	2.1%	12.1%	100.0%
		% within contact made with water	100.0%	100.0%	100.0%	100.0 %	100.0%	100.0%
		% of Total	22.1%	55.5%	8.2%	2.1%	12.1%	100.0%

Chi-Square Tests

	Value	df	Asymp. Sig. (2-sided)
Pearson Chi-Square	37.678[a]	12	.000
Likelihood Ratio	35.077	12	.000
Linear-by-Linear Association	1.086	1	.297
N of Valid Cases	620		

a. 5 cells (25.0%) have expected count less than 5. The minimum expected count is .30.

prevention of infection * contact made with water

Chi-Square Tests

	Value	df	Asymp. Sig. (2-sided)
Pearson Chi-Square	32.813[a]	16	.008
Likelihood Ratio	32.874	16	.008
Linear-by-Linear Association	3.662	1	.056
N of Valid Cases	620		

a. 5 cells (20.0%) have expected count less than 5. The minimum expected count is 1.32.

Crosstab

			mother complete primary education		Total
			yes	no	
prevention of infection	stop going to river	Count	64	16	80
		Expected Count	55.7	24.3	80.0
		% within prevention of infection	80.0%	20.0%	100.0%
		% within mother complete primary education	14.8%	8.5%	12.9%
		% of Total	10.3%	2.6%	12.9%
	eating good food	Count	123	65	188
		Expected Count	131.0	57.0	188.0
		% within prevention of infection	65.4%	34.6%	100.0%
		% within mother complete primary education	28.5%	34.6%	30.3%
		% of Total	19.8%	10.5%	30.3%
	bathing regularly	Count	34	29	63
		Expected Count	43.9	19.1	63.0
		% within prevention of infection	54.0%	46.0%	100.0%
		% within mother complete primary education	7.9%	15.4%	10.2%
		% of Total	5.5%	4.7%	10.2%
	treat drinking water	Count	139	67	206
		Expected Count	143.5	62.5	206.0
		% within prevention of infection	67.5%	32.5%	100.0%

		% within mother complete primary education	32.2%	35.6%	33.2%
		% of Total	22.4%	10.8%	33.2%
	i don'know	Count	72	11	83
		Expected Count	57.8	25.2	83.0
		% within prevention of infection	86.7%	13.3%	100.0%
		% within mother complete primary education	16.7%	5.9%	13.4%
		% of Total	11.6%	1.8%	13.4%
Total		Count	432	188	620
		Expected Count	432.0	188.0	620.0
		% within prevention of infection	69.7%	30.3%	100.0%
		% within mother complete primary education	100.0%	100.0%	100.0%
		% of Total	69.7%	30.3%	100.0%

prevention of infection * mother complete primary education

Chi-Square Tests

	Value	df	Asymp. Sig. (2-sided)
Pearson Chi-Square	24.921[a]	4	.000
Likelihood Ratio	26.586	4	.000
Linear-by-Linear Association	1.055	1	.304
N of Valid Cases	620		

a. 0 cells (.0%) have expected count less than 5. The minimum expected count is 19.10.

prevention of infection * father complete primary education

Crosstab

			father complete primary education		Total
			yes	no	
prevention of infection	stop going to river	Count	73	7	80
		Expected Count	61.5	18.5	80.0
		% within prevention of infection	91.2%	8.8%	100.0%
		% within father complete primary education	15.3%	4.9%	12.9%
		% of Total	11.8%	1.1%	12.9%
	eating good food	Count	140	48	188
		Expected Count	144.6	43.4	188.0
		% within prevention of infection	74.5%	25.5%	100.0%
		% within father complete primary education	29.4%	33.6%	30.3%
		% of Total	22.6%	7.7%	30.3%
	bathing regularly	Count	34	29	63
		Expected Count	48.5	14.5	63.0
		% within prevention of infection	54.0%	46.0%	100.0%
		% within father complete primary education	7.1%	20.3%	10.2%
		% of Total	5.5%	4.7%	10.2%
	treat drinking water	Count	153	53	206
		Expected Count	158.5	47.5	206.0
		% within prevention of infection	74.3%	25.7%	100.0%

		% within father complete primary education	32.1%	37.1%	33.2%
		% of Total	24.7%	8.5%	33.2%
	i don'know	Count	77	6	83
		Expected Count	63.9	19.1	83.0
		% within prevention of infection	92.8%	7.2%	100.0%
		% within father complete primary education	16.1%	4.2%	13.4%
		% of Total	12.4%	1.0%	13.4%
Total		Count	477	143	620
		Expected Count	477.0	143.0	620.0
		% within prevention of infection	76.9%	23.1%	100.0%
		% within father complete primary education	100.0%	100.0%	100.0%
		% of Total	76.9%	23.1%	100.0%

Chi-Square Tests

	Value	df	Asymp. Sig. (2-sided)
Pearson Chi-Square	41.164[a]	4	.000
Likelihood Ratio	43.647	4	.000
Linear-by-Linear Association	.035	1	.852
N of Valid Cases	620		

a. 0 cells (.0%) have expected count less than 5. The minimum expected count is 14.53.

Crosstab

			mother's occupation				Total
			trading	farming	salary earner	unemployed	
prevention of infection	stop going to river	Count	52	11	13	4	80
		Expected Count	37.5	17.8	16.6	8.0	80.0
		% within prevention of infection	65.0%	13.8%	16.2%	5.0%	100.0%
		% within mother's occupation	17.9%	8.0%	10.1%	6.5%	12.9%
		% of Total	8.4%	1.8%	2.1%	.6%	12.9%
	eating good food	Count	83	49	35	21	188
		Expected Count	88.2	41.8	39.1	18.8	188.0
		% within prevention of infection	44.1%	26.1%	18.6%	11.2%	100.0%
		% within mother's occupation	28.5%	35.5%	27.1%	33.9%	30.3%
		% of Total	13.4%	7.9%	5.6%	3.4%	30.3%
	bathing regularly	Count	22	27	11	3	63
		Expected Count	29.6	14.0	13.1	6.3	63.0
		% within prevention of infection	34.9%	42.9%	17.5%	4.8%	100.0%
		% within mother's occupation	7.6%	19.6%	8.5%	4.8%	10.2%
		% of Total	3.5%	4.4%	1.8%	.5%	10.2%
	treat drinking water	Count	86	46	44	30	206
		Expected Count	96.7	45.9	42.9	20.6	206.0

		% within prevention of infection	41.7%	22.3%	21.4%	14.6%	100.0%
		% within mother's occupation	29.6%	33.3%	34.1%	48.4%	33.2%
		% of Total	13.9%	7.4%	7.1%	4.8%	33.2%
	i don't know	Count	48	5	26	4	83
		Expected Count	39.0	18.5	17.3	8.3	83.0
		% within prevention of infection	57.8%	6.0%	31.3%	4.8%	100.0%
		% within mother's occupation	16.5%	3.6%	20.2%	6.5%	13.4%
		% of Total	7.7%	.8%	4.2%	.6%	13.4%
Total		Count	291	138	129	62	620
		Expected Count	291.0	138.0	129.0	62.0	620.0
		% within prevention of infection	46.9%	22.3%	20.8%	10.0%	100.0%
		% within mother's occupation	100.0%	100.0%	100.0%	100.0%	100.0%
		% of Total	46.9%	22.3%	20.8%	10.0%	100.0%

prevention of infection * mother's occupation

Crosstab

			father's occupation					
			fishing	farming	trading	salary earner	wage earner	Total
prevention of infection	stop going to river	Count	2	29	27	19	3	80
		Expected Count	1.9	33.9	20.8	17.0	6.3	80.0
		% within prevention of infection	2.5%	36.2%	33.8%	23.8%	3.8%	100.0%
		% within father's occupation	13.3%	11.0%	16.8%	14.4%	6.1%	12.9%
		% of Total	.3%	4.7%	4.4%	3.1%	.5%	12.9%
	eating good food	Count	8	80	41	40	19	188
		Expected Count	4.5	79.7	48.8	40.0	14.9	188.0
		% within prevention of infection	4.3%	42.6%	21.8%	21.3%	10.1%	100.0%
		% within father's occupation	53.3%	30.4%	25.5%	30.3%	38.8%	30.3%
		% of Total	1.3%	12.9%	6.6%	6.5%	3.1%	30.3%
	bathing regularly	Count	3	41	11	5	3	63
		Expected Count	1.5	26.7	16.4	13.4	5.0	63.0
		% within prevention of infection	4.8%	65.1%	17.5%	7.9%	4.8%	100.0%
		% within father's occupation	20.0%	15.6%	6.8%	3.8%	6.1%	10.2%
		% of Total	.5%	6.6%	1.8%	.8%	.5%	10.2%
	treat drinking water	Count	2	86	56	44	18	206
		Expected Count	5.0	87.4	53.5	43.9	16.3	206.0
		% within prevention of infection	1.0%	41.7%	27.2%	21.4%	8.7%	100.0%

		% within father's occupation	13.3%	32.7%	34.8%	33.3%	36.7%	33.2%
		% of Total	.3%	13.9%	9.0%	7.1%	2.9%	33.2%
	i don'know	Count	0	27	26	24	6	83
		Expected Count	2.0	35.2	21.6	17.7	6.6	83.0
		% within prevention of infection	.0%	32.5%	31.3%	28.9%	7.2%	100.0%
		% within father's occupation	.0%	10.3%	16.1%	18.2%	12.2%	13.4%
		% of Total	.0%	4.4%	4.2%	3.9%	1.0%	13.4%
Total		Count	15	263	161	132	49	620
		Expected Count	15.0	263.0	161.0	132.0	49.0	620.0
		% within prevention of infection	2.4%	42.4%	26.0%	21.3%	7.9%	100.0%
		% within father's occupation	100.0%	100.0%	100.0%	100.0%	100.0%	100.0%
		% of Total	2.4%	42.4%	26.0%	21.3%	7.9%	100.0%

Chi-Square Tests

	Value	df	Asymp. Sig. (2-sided)
Pearson Chi-Square	53.272[a]	12	.000
Likelihood Ratio	54.949	12	.000
Linear-by-Linear Association	3.147	1	.076
N of Valid Cases	620		

Chi-Square Tests

	Value	df	Asymp. Sig. (2-sided)
Pearson Chi-Square	53.272[a]	12	.000
Likelihood Ratio	54.949	12	.000
Linear-by-Linear Association	3.147	1	.076

a. 0 cells (.0%) have expected count less than 5. The minimum expected count is 6.30.

prevention of infection * father's occupation

Chi-Square Tests

	Value	df	Asymp. Sig. (2-sided)
Pearson Chi-Square	35.722[a]	16	.003
Likelihood Ratio	38.562	16	.001
Linear-by-Linear Association	1.815	1	.178
N of Valid Cases	620		

a. 6 cells (24.0%) have expected count less than 5. The minimum expected count is 1.52.

prevention of infection * sex

Crosstab

			sex		Total
			male	female	
prevention of infection	stop going to river	Count	48	32	80
		Expected Count	46.7	33.3	80.0
		% within prevention of infection	60.0%	40.0%	100.0%
		% within sex	13.3%	12.4%	12.9%
		% of Total	7.7%	5.2%	12.9%
	eating good food	Count	110	78	188
		Expected Count	109.8	78.2	188.0
		% within prevention of infection	58.5%	41.5%	100.0%
		% within sex	30.4%	30.2%	30.3%
		% of Total	17.7%	12.6%	30.3%
	bathing regularly	Count	42	21	63
		Expected Count	36.8	26.2	63.0
		% within prevention of infection	66.7%	33.3%	100.0%
		% within sex	11.6%	8.1%	10.2%
		% of Total	6.8%	3.4%	10.2%
	treat drinking water	Count	118	88	206
		Expected Count	120.3	85.7	206.0
		% within prevention of infection	57.3%	42.7%	100.0%
		% within sex	32.6%	34.1%	33.2%
		% of Total	19.0%	14.2%	33.2%

	i don'know	Count	44	39	83
		Expected Count	48.5	34.5	83.0
		% within prevention of infection	53.0%	47.0%	100.0%
		% within sex	12.2%	15.1%	13.4%
		% of Total	7.1%	6.3%	13.4%
Total		Count	362	258	620
		Expected Count	362.0	258.0	620.0
		% within prevention of infection	58.4%	41.6%	100.0%
		% within sex	100.0%	100.0%	100.0%
		% of Total	58.4%	41.6%	100.0%

Chi-Square Tests

	Value	df	Asymp. Sig. (2-sided)
Pearson Chi-Square	2.955[a]	4	.565
Likelihood Ratio	2.989	4	.560
Linear-by-Linear Association	.772	1	.380
N of Valid Cases	620		

a. 0 cells (.0%) have expected count less than 5. The minimum expected count is 26.22.

prevention of infection * Age distribution

Crosstab

			Age distribution					Total
			<_4	5 - 9	10 - 14	15 - 19	>_20	
prevention of infection	stop going to river	Count	7	6	41	26	0	80
		Expected Count	2.2	20.1	34.8	21.7	1.2	80.0
		% within prevention of infection	8.8%	7.5%	51.2%	32.5%	.0%	100.0%
		% within Age distribution	41.2%	3.8%	15.2%	15.5%	.0%	12.9%
		% of Total	1.1%	1.0%	6.6%	4.2%	.0%	12.9%
	eating good food	Count	4	55	76	52	1	188
		Expected Count	5.2	47.3	81.9	50.9	2.7	188.0
		% within prevention of infection	2.1%	29.3%	40.4%	27.7%	.5%	100.0%
		% within Age distribution	23.5%	35.3%	28.1%	31.0%	11.1%	30.3%
		% of Total	.6%	8.9%	12.3%	8.4%	.2%	30.3%
	bathing regularly	Count	2	14	30	15	2	63
		Expected Count	1.7	15.9	27.4	17.1	.9	63.0
		% within prevention of infection	3.2%	22.2%	47.6%	23.8%	3.2%	100.0%
		% within Age distribution	11.8%	9.0%	11.1%	8.9%	22.2%	10.2%
		% of Total	.3%	2.3%	4.8%	2.4%	.3%	10.2%
	treat drinking water	Count	4	65	81	52	4	206
		Expected Count	5.6	51.8	89.7	55.8	3.0	206.0
		% within prevention of infection	1.9%	31.6%	39.3%	25.2%	1.9%	100.0%

		% within Age distribution	23.5%	41.7%	30.0%	31.0%	44.4%	33.2%
		% of Total	.6%	10.5%	13.1%	8.4%	.6%	33.2%
	i don'know	Count	0	16	42	23	2	83
		Expected Count	2.3	20.9	36.1	22.5	1.2	83.0
		% within prevention of infection	.0%	19.3%	50.6%	27.7%	2.4%	100.0%
		% within Age distribution	.0%	10.3%	15.6%	13.7%	22.2%	13.4%
		% of Total	.0%	2.6%	6.8%	3.7%	.3%	13.4%
Total		Count	17	156	270	168	9	620
		Expected Count	17.0	156.0	270.0	168.0	9.0	620.0
		% within prevention of infection	2.7%	25.2%	43.5%	27.1%	1.5%	100.0%
		% within Age distribution	100.0%	100.0%	100.0%	100.0%	100.0%	100.0%
		% of Total	2.7%	25.2%	43.5%	27.1%	1.5%	100.0%

Crosstab

			main source of water supply				Total
			tap	well	river	others	
symtoms of infection	blood in urine	Count	17	99	96	1	213
		Expected Count	24.4	106.2	81.4	1.0	213.0
		% within symtoms of infection	8.0%	46.5%	45.1%	.5%	100.0%
		% within main source of water supply	23.9%	32.0%	40.5%	33.3%	34.4%
		% of Total	2.7%	16.0%	15.5%	.2%	34.4%
	stomach pain	Count	29	115	87	0	231
		Expected Count	26.5	115.1	88.3	1.1	231.0
		% within symtoms of infection	12.6%	49.8%	37.7%	.0%	100.0%
		% within main source of water supply	40.8%	37.2%	36.7%	.0%	37.3%
		% of Total	4.7%	18.5%	14.0%	.0%	37.3%
	waist pain	Count	9	57	23	0	89
		Expected Count	10.2	44.4	34.0	.4	89.0
		% within symtoms of infection	10.1%	64.0%	25.8%	.0%	100.0%
		% within main source of water supply	12.7%	18.4%	9.7%	.0%	14.4%
		% of Total	1.5%	9.2%	3.7%	.0%	14.4%
	blurred vision	Count	6	9	12	0	27
		Expected Count	3.1	13.5	10.3	.1	27.0
		% within symtoms of infection	22.2%	33.3%	44.4%	.0%	100.0%
		% within main source of water supply	8.5%	2.9%	5.1%	.0%	4.4%
		% of Total	1.0%	1.5%	1.9%	.0%	4.4%
	i don't know	Count	10	29	19	2	60

	Expected Count	6.9	29.9	22.9	.3	60.0
	% within symtoms of infection	16.7%	48.3%	31.7%	3.3%	100.0%
	% within main source of water supply	14.1%	9.4%	8.0%	66.7%	9.7%
	% of Total	1.6%	4.7%	3.1%	.3%	9.7%
Total	Count	71	309	237	3	620
	Expected Count	71.0	309.0	237.0	3.0	620.0
	% within symtoms of infection	11.5%	49.8%	38.2%	.5%	100.0%
	% within main source of water supply	100.0%	100.0%	100.0%	100.0%	100.0%
	% of Total	11.5%	49.8%	38.2%	.5%	100.0%

Chi-Square Tests

	Value	df	Asymp. Sig. (2-sided)
Pearson Chi-Square	38.827[a]	16	.001
Likelihood Ratio	41.845	16	.000
Linear-by-Linear Association	.052	1	.819
N of Valid Cases	620		

a. 8 cells (32.0%) have expected count less than 5. The minimum expected count is .91.

symtoms of infection * main source of water supply

Chi-Square Tests

	Value	df	Asymp. Sig. (2-sided)
Pearson Chi-Square	31.271[a]	12	.002
Likelihood Ratio	26.932	12	.008
Linear-by-Linear Association	5.286	1	.021
N of Valid Cases	620		

a. 6 cells (30.0%) have expected count less than 5. The minimum expected count is .13.

Crosstab

			contact made with water					Total
			play or bath	washing	agricultural work	fishing	no contact	
symtoms of infection	blood in urine	Count	40	119	22	7	25	213
		Expected Count	47.1	118.2	17.5	4.5	25.8	213.0
		% within symtoms of infection	18.8%	55.9%	10.3%	3.3%	11.7%	100.0%
		% within contact made with water	29.2%	34.6%	43.1%	53.8%	33.3%	34.4%
		% of Total	6.5%	19.2%	3.5%	1.1%	4.0%	34.4%
	stomach pain	Count	48	144	9	5	25	231
		Expected Count	51.0	128.2	19.0	4.8	27.9	231.0
		% within symtoms of infection	20.8%	62.3%	3.9%	2.2%	10.8%	100.0%
		% within contact made with water	35.0%	41.9%	17.6%	38.5%	33.3%	37.3%
		% of Total	7.7%	23.2%	1.5%	.8%	4.0%	37.3%
	waist pain	Count	20	45	10	1	13	89
		Expected Count	19.7	49.4	7.3	1.9	10.8	89.0
		% within symtoms of infection	22.5%	50.6%	11.2%	1.1%	14.6%	100.0%
		% within contact made with water	14.6%	13.1%	19.6%	7.7%	17.3%	14.4%
		% of Total	3.2%	7.3%	1.6%	.2%	2.1%	14.4%
		Count	10	9	5	0	3	27

	blurred vision	Expected Count	6.0	15.0	2.2	.6	3.3	27.0
		% within symtoms of infection	37.0%	33.3%	18.5%	.0%	11.1%	100.0%
		% within contact made with water	7.3%	2.6%	9.8%	.0%	4.0%	4.4%
		% of Total	1.6%	1.5%	.8%	.0%	.5%	4.4%
	i don't know	Count	19	27	5	0	9	60
		Expected Count	13.3	33.3	4.9	1.3	7.3	60.0
		% within symtoms of infection	31.7%	45.0%	8.3%	.0%	15.0%	100.0%
		% within contact made with water	13.9%	7.8%	9.8%	.0%	12.0%	9.7%
		% of Total	3.1%	4.4%	.8%	.0%	1.5%	9.7%
Total		Count	137	344	51	13	75	620
		Expected Count	137.0	344.0	51.0	13.0	75.0	620.0
		% within symtoms of infection	22.1%	55.5%	8.2%	2.1%	12.1%	100.0%
		% within contact made with water	100.0%	100.0%	100.0%	100.0%	100.0%	100.0%
		% of Total	22.1%	55.5%	8.2%	2.1%	12.1%	100.0%

symtoms of infection * contact made with water

Chi-Square Tests

	Value	df	Asymp. Sig. (2-sided)
Pearson Chi-Square	28.161[a]	16	.030
Likelihood Ratio	29.706	16	.020
Linear-by-Linear Association	.379	1	.538
N of Valid Cases	620		

a. 8 cells (32.0%) have expected count less than 5. The minimum expected count is .57.

symtoms of infection * mother complete primary education

Crosstab

			mother complete primary education		Total
			yes	no	
symtoms of infection	blood in urine	Count	149	64	213
		Expected Count	148.4	64.6	213.0
		% within symtoms of infection	70.0%	30.0%	100.0%
		% within mother complete primary education	34.5%	34.0%	34.4%
		% of Total	24.0%	10.3%	34.4%
	stomach pain	Count	155	76	231
		Expected Count	161.0	70.0	231.0
		% within symtoms of infection	67.1%	32.9%	100.0%
		% within mother complete primary education	35.9%	40.4%	37.3%
		% of Total	25.0%	12.3%	37.3%
	waist pain	Count	62	27	89
		Expected Count	62.0	27.0	89.0
		% within symtoms of infection	69.7%	30.3%	100.0%
		% within mother complete primary education	14.4%	14.4%	14.4%
		% of Total	10.0%	4.4%	14.4%
	blurred vision	Count	15	12	27
		Expected Count	18.8	8.2	27.0
		% within symtoms of infection	55.6%	44.4%	100.0%

		% within mother complete primary education	3.5%	6.4%	4.4%
		% of Total	2.4%	1.9%	4.4%
	i don't know	Count	51	9	60
		Expected Count	41.8	18.2	60.0
		% within symtoms of infection	85.0%	15.0%	100.0%
		% within mother complete primary education	11.8%	4.8%	9.7%
		% of Total	8.2%	1.5%	9.7%
Total		Count	432	188	620
		Expected Count	432.0	188.0	620.0
		% within symtoms of infection	69.7%	30.3%	100.0%
		% within mother complete primary education	100.0%	100.0%	100.0%
		% of Total	69.7%	30.3%	100.0%

Chi-Square Tests

	Value	df	Asymp. Sig. (2-sided)
Pearson Chi-Square	9.950[a]	4	.041
Likelihood Ratio	10.711	4	.030
Linear-by-Linear Association	1.902	1	.168
N of Valid Cases	620		

a. 0 cells (.0%) have expected count less than 5. The minimum expected count is 8.19.

Crosstab

			father complete primary education		
			yes	no	Total
symtoms of infection	blood in urine	Count	163	50	213
		Expected Count	163.9	49.1	213.0
		% within symtoms of infection	76.5%	23.5%	100.0%
		% within father complete primary education	34.2%	35.0%	34.4%
		% of Total	26.3%	8.1%	34.4%
	stomach pain	Count	171	60	231
		Expected Count	177.7	53.3	231.0
		% within symtoms of infection	74.0%	26.0%	100.0%
		% within father complete primary education	35.8%	42.0%	37.3%
		% of Total	27.6%	9.7%	37.3%
	waist pain	Count	73	16	89
		Expected Count	68.5	20.5	89.0
		% within symtoms of infection	82.0%	18.0%	100.0%
		% within father complete primary education	15.3%	11.2%	14.4%
		% of Total	11.8%	2.6%	14.4%
	blurred vision	Count	14	13	27
		Expected Count	20.8	6.2	27.0
		% within symtoms of infection	51.9%	48.1%	100.0%
		% within father complete primary education	2.9%	9.1%	4.4%

		% of Total	2.3%	2.1%	4.4%
	i don't know	Count	56	4	60
		Expected Count	46.2	13.8	60.0
		% within symtoms of infection	93.3%	6.7%	100.0%
		% within father complete primary education	11.7%	2.8%	9.7%
		% of Total	9.0%	.6%	9.7%
Total		Count	477	143	620
		Expected Count	477.0	143.0	620.0
		% within symtoms of infection	76.9%	23.1%	100.0%
		% within father complete primary education	100.0%	100.0%	100.0%
		% of Total	76.9%	23.1%	100.0%

symtoms of infection * father complete primary education

Crosstab

			mother's occupation				Total
			trading	farming	salary earner	unemployed	
symtoms of infection	blood in urine	Count	95	58	34	26	213
		Expected Count	100.0	47.4	44.3	21.3	213.0
		% within symtoms of infection	44.6%	27.2%	16.0%	12.2%	100.0%
		% within mother's occupation	32.6%	42.0%	26.4%	41.9%	34.4%
		% of Total	15.3%	9.4%	5.5%	4.2%	34.4%
	stomach pain	Count	115	55	42	19	231
		Expected Count	108.4	51.4	48.1	23.1	231.0
		% within symtoms of infection	49.8%	23.8%	18.2%	8.2%	100.0%
		% within mother's occupation	39.5%	39.9%	32.6%	30.6%	37.3%
		% of Total	18.5%	8.9%	6.8%	3.1%	37.3%
	waist pain	Count	36	15	27	11	89
		Expected Count	41.8	19.8	18.5	8.9	89.0
		% within symtoms of infection	40.4%	16.9%	30.3%	12.4%	100.0%
		% within mother's occupation	12.4%	10.9%	20.9%	17.7%	14.4%
		% of Total	5.8%	2.4%	4.4%	1.8%	14.4%
	blurred vision	Count	11	7	7	2	27
		Expected Count	12.7	6.0	5.6	2.7	27.0
		% within symtoms of infection	40.7%	25.9%	25.9%	7.4%	100.0%
		% within mother's occupation	3.8%	5.1%	5.4%	3.2%	4.4%

		% of Total	1.8%	1.1%	1.1%	.3%	4.4%
	i don't know	Count	34	3	19	4	60
		Expected Count	28.2	13.4	12.5	6.0	60.0
		% within symtoms of infection	56.7%	5.0%	31.7%	6.7%	100.0%
		% within mother's occupation	11.7%	2.2%	14.7%	6.5%	9.7%
		% of Total	5.5%	.5%	3.1%	.6%	9.7%
Total		Count	291	138	129	62	620
		Expected Count	291.0	138.0	129.0	62.0	620.0
		% within symtoms of infection	46.9%	22.3%	20.8%	10.0%	100.0%
		% within mother's occupation	100.0%	100.0%	100.0%	100.0%	100.0%
		% of Total	46.9%	22.3%	20.8%	10.0%	100.0%

Chi-Square Tests

	Value	df	Asymp. Sig. (2-sided)
Pearson Chi-Square	21.085[a]	4	.000
Likelihood Ratio	22.264	4	.000
Linear-by-Linear Association	2.762	1	.097
N of Valid Cases	620		

a. 0 cells (.0%) have expected count less than 5. The minimum expected count is 6.23.

Crosstab

			father's occupation					
			fishing	farming	trading	salary earner	wage earner	Total
symtoms of infection	blood in urine	Count	11	92	55	42	13	213
		Expected Count	5.2	90.4	55.3	45.3	16.8	213.0
		% within symtoms of infection	5.2%	43.2%	25.8%	19.7%	6.1%	100.0%
		% within father's occupation	73.3%	35.0%	34.2%	31.8%	26.5%	34.4%
		% of Total	1.8%	14.8%	8.9%	6.8%	2.1%	34.4%
	stomach pain	Count	4	104	62	44	17	231
		Expected Count	5.6	98.0	60.0	49.2	18.3	231.0
		% within symtoms of infection	1.7%	45.0%	26.8%	19.0%	7.4%	100.0%
		% within father's occupation	26.7%	39.5%	38.5%	33.3%	34.7%	37.3%
		% of Total	.6%	16.8%	10.0%	7.1%	2.7%	37.3%
	waist pain	Count	0	36	19	25	9	89
		Expected Count	2.2	37.8	23.1	18.9	7.0	89.0
		% within symtoms of infection	.0%	40.4%	21.3%	28.1%	10.1%	100.0%
		% within father's occupation	.0%	13.7%	11.8%	18.9%	18.4%	14.4%
		% of Total	.0%	5.8%	3.1%	4.0%	1.5%	14.4%
	blurred vision	Count	0	14	8	4	1	27
		Expected Count	.7	11.5	7.0	5.7	2.1	27.0

		% within symtoms of infection	.0%	51.9%	29.6%	14.8%	3.7%	100.0%
		% within father's occupation	.0%	5.3%	5.0%	3.0%	2.0%	4.4%
		% of Total	.0%	2.3%	1.3%	.6%	.2%	4.4%
	i don't know	Count	0	17	17	17	9	60
		Expected Count	1.5	25.5	15.6	12.8	4.7	60.0
		% within symtoms of infection	.0%	28.3%	28.3%	28.3%	15.0%	100.0%
		% within father's occupation	.0%	6.5%	10.6%	12.9%	18.4%	9.7%
		% of Total	.0%	2.7%	2.7%	2.7%	1.5%	9.7%
Total		Count	15	263	161	132	49	620
		Expected Count	15.0	263.0	161.0	132.0	49.0	620.0
		% within symtoms of infection	2.4%	42.4%	26.0%	21.3%	7.9%	100.0%
		% within father's occupation	100.0%	100.0%	100.0%	100.0%	100.0%	100.0%
		% of Total	2.4%	42.4%	26.0%	21.3%	7.9%	100.0%

symtoms of infection * mother's occupation

Chi-Square Tests

	Value	df	Asymp. Sig. (2-sided)
Pearson Chi-Square	28.753[a]	12	.004
Likelihood Ratio	31.693	12	.002
Linear-by-Linear Association	.060	1	.807
N of Valid Cases	620		

a. 1 cells (5.0%) have expected count less than 5. The minimum expected count is 2.70.

symtoms of infection * father's occupation

Chi-Square Tests

	Value	df	Asymp. Sig. (2-sided)
Pearson Chi-Square	26.857[a]	16	.043
Likelihood Ratio	29.043	16	.024
Linear-by-Linear Association	10.775	1	.001
N of Valid Cases	620		

a. 5 cells (20.0%) have expected count less than 5. The minimum expected count is .65.

symtoms of infection * sex

Crosstab

			sex		Total
			male	female	
symtoms of infection	blood in urine	Count	132	81	213
		Expected Count	124.4	88.6	213.0
		% within symtoms of infection	62.0%	38.0%	100.0%
		% within sex	36.5%	31.4%	34.4%
		% of Total	21.3%	13.1%	34.4%
	stomach pain	Count	130	101	231
		Expected Count	134.9	96.1	231.0
		% within symtoms of infection	56.3%	43.7%	100.0%
		% within sex	35.9%	39.1%	37.3%
		% of Total	21.0%	16.3%	37.3%
	waist pain	Count	48	41	89
		Expected Count	52.0	37.0	89.0
		% within symtoms of infection	53.9%	46.1%	100.0%
		% within sex	13.3%	15.9%	14.4%
		% of Total	7.7%	6.6%	14.4%
	blurred vision	Count	18	9	27
		Expected Count	15.8	11.2	27.0
		% within symtoms of infection	66.7%	33.3%	100.0%
		% within sex	5.0%	3.5%	4.4%

		% of Total	2.9%	1.5%	4.4%
	i don't know	Count	34	26	60
		Expected Count	35.0	25.0	60.0
		% within symtoms of infection	56.7%	43.3%	100.0%
		% within sex	9.4%	10.1%	9.7%
		% of Total	5.5%	4.2%	9.7%
Total		Count	362	258	620
		Expected Count	362.0	258.0	620.0
		% within symtoms of infection	58.4%	41.6%	100.0%
		% within sex	100.0%	100.0%	100.0%
		% of Total	58.4%	41.6%	100.0%

Crosstab

			Age distribution					Total
			<_4	5 - 9	10 - 14	15 - 19	>_20	
symtoms of infection	blood in urine	Count	6	56	88	62	1	213
		Expected Count	5.8	53.6	92.8	57.7	3.1	213.0
		% within symtoms of infection	2.8%	26.3%	41.3%	29.1%	.5%	100.0%
		% within Age distribution	35.3%	35.9%	32.6%	36.9%	11.1%	34.4%
		% of Total	1.0%	9.0%	14.2%	10.0%	.2%	34.4%
	stomach pain	Count	10	50	112	53	6	231
		Expected Count	6.3	58.1	100.6	62.6	3.4	231.0
		% within symtoms of infection	4.3%	21.6%	48.5%	22.9%	2.6%	100.0%
		% within Age distribution	58.8%	32.1%	41.5%	31.5%	66.7%	37.3%
		% of Total	1.6%	8.1%	18.1%	8.5%	1.0%	37.3%
	waist pain	Count	1	31	34	23	0	89
		Expected Count	2.4	22.4	38.8	24.1	1.3	89.0
		% within symtoms of infection	1.1%	34.8%	38.2%	25.8%	.0%	100.0%
		% within Age distribution	5.9%	19.9%	12.6%	13.7%	.0%	14.4%
		% of Total	.2%	5.0%	5.5%	3.7%	.0%	14.4%
	blurred vision	Count	0	4	12	11	0	27
		Expected Count	.7	6.8	11.8	7.3	.4	27.0
		% within symtoms of infection	.0%	14.8%	44.4%	40.7%	.0%	100.0%
		% within Age distribution	.0%	2.6%	4.4%	6.5%	.0%	4.4%
		% of Total	.0%	.6%	1.9%	1.8%	.0%	4.4%

	i don't know	Count	0	15	24	19	2	60
		Expected Count	1.6	15.1	26.1	16.3	.9	60.0
		% within symtoms of infection	.0%	25.0%	40.0%	31.7%	3.3%	100.0%
		% within Age distribution	.0%	9.6%	8.9%	11.3%	22.2%	9.7%
		% of Total	.0%	2.4%	3.9%	3.1%	.3%	9.7%
Total		Count	17	156	270	168	9	620
		Expected Count	17.0	156.0	270.0	168.0	9.0	620.0
		% within symtoms of infection	2.7%	25.2%	43.5%	27.1%	1.5%	100.0%
		% within Age distribution	100.0 %	100.0%	100.0%	100.0%	100.0%	100.0%
		% of Total	2.7%	25.2%	43.5%	27.1%	1.5%	100.0%

Chi-Square Tests

	Value	df	Asymp. Sig. (2-sided)
Pearson Chi-Square	3.112[a]	4	.539
Likelihood Ratio	3.132	4	.536
Linear-by-Linear Association	.462	1	.497
N of Valid Cases	620		

a. 0 cells (.0%) have expected count less than 5. The minimum expected count is 11.24.

symtoms of infection * Age distribution

Crosstab

			main source of water supply				Total
			tap	well	river	others	Total
intermidiate host	housefly and cocroaches	Count	23	139	120	0	282
		Expected Count	32.3	140.5	107.8	1.4	282.0
		% within intermidiate host	8.2%	49.3%	42.6%	.0%	100.0%
		% within main source of water supply	32.4%	45.0%	50.6%	.0%	45.5%
		% of Total	3.7%	22.4%	19.4%	.0%	45.5%
	water snail	Count	20	51	27	1	99
		Expected Count	11.3	49.3	37.8	.5	99.0
		% within intermidiate host	20.2%	51.5%	27.3%	1.0%	100.0%
		% within main source of water supply	28.2%	16.5%	11.4%	33.3%	16.0%
		% of Total	3.2%	8.2%	4.4%	.2%	16.0%
	fish	Count	14	60	61	0	135
		Expected Count	15.5	67.3	51.6	.7	135.0
		% within intermidiate host	10.4%	44.4%	45.2%	.0%	100.0%
		% within main source of water supply	19.7%	19.4%	25.7%	.0%	21.8%
		% of Total	2.3%	9.7%	9.8%	.0%	21.8%
	houserat	Count	7	31	12	1	51
		Expected Count	5.8	25.4	19.5	.2	51.0
		% within intermidiate host	13.7%	60.8%	23.5%	2.0%	100.0%
		% within main source of water supply	9.9%	10.0%	5.1%	33.3%	8.2%
		% of Total	1.1%	5.0%	1.9%	.2%	8.2%

	i don't know	Count	7	28	17	1	53
		Expected Count	6.1	26.4	20.3	.3	53.0
		% within intermidiate host	13.2%	52.8%	32.1%	1.9%	100.0%
		% within main source of water supply	9.9%	9.1%	7.2%	33.3%	8.5%
		% of Total	1.1%	4.5%	2.7%	.2%	8.5%
Total		Count	71	309	237	3	620
		Expected Count	71.0	309.0	237.0	3.0	620.0
		% within intermidiate host	11.5%	49.8%	38.2%	.5%	100.0%
		% within main source of water supply	100.0%	100.0%	100.0%	100.0%	100.0%
		% of Total	11.5%	49.8%	38.2%	.5%	100.0%

Chi-Square Tests

	Value	df	Asymp. Sig. (2-sided)
Pearson Chi-Square	24.171[a]	16	.086
Likelihood Ratio	27.567	16	.036
Linear-by-Linear Association	1.663	1	.197
N of Valid Cases	620		

a. 8 cells (32.0%) have expected count less than 5. The minimum expected count is .39.

intermidiate host * main source of water supply

Chi-Square Tests

	Value	df	Asymp. Sig. (2-sided)
Pearson Chi-Square	28.631[a]	12	.004
Likelihood Ratio	28.274	12	.005
Linear-by-Linear Association	2.111	1	.146
N of Valid Cases	620		

a. 5 cells (25.0%) have expected count less than 5. The minimum expected count is .25.

Crosstab

			contact made with water					
			play or bath	washing	agricultural work	fishing	no contact	Total
intermidiate host	housefly and cocroaches	Count	55	172	20	6	29	282
		Expected Count	62.3	156.5	23.2	5.9	34.1	282.0
		% within intermidiate host	19.5%	61.0%	7.1%	2.1%	10.3%	100.0%
		% within contact made with water	40.1%	50.0%	39.2%	46.2%	38.7%	45.5%
		% of Total	8.9%	27.7%	3.2%	1.0%	4.7%	45.5%
	water snail	Count	23	57	6	1	12	99
		Expected Count	21.9	54.9	8.1	2.1	12.0	99.0
		% within intermidiate host	23.2%	57.6%	6.1%	1.0%	12.1%	100.0%
		% within contact made with water	16.8%	16.6%	11.8%	7.7%	16.0%	16.0%
		% of Total	3.7%	9.2%	1.0%	.2%	1.9%	16.0%
	fish	Count	24	74	16	4	17	135
		Expected Count	29.8	74.9	11.1	2.8	16.3	135.0
		% within intermidiate host	17.8%	54.8%	11.9%	3.0%	12.6%	100.0%
		% within contact made with water	17.5%	21.5%	31.4%	30.8%	22.7%	21.8%
		% of Total	3.9%	11.9%	2.6%	.6%	2.7%	21.8%

	houserat	Count	17	20	2	1	11	51
		Expected Count	11.3	28.3	4.2	1.1	6.2	51.0
		% within intermidiate host	33.3%	39.2%	3.9%	2.0%	21.6%	100.0%
		% within contact made with water	12.4%	5.8%	3.9%	7.7%	14.7%	8.2%
		% of Total	2.7%	3.2%	.3%	.2%	1.8%	8.2%
	i don't know	Count	18	21	7	1	6	53
		Expected Count	11.7	29.4	4.4	1.1	6.4	53.0
		% within intermidiate host	34.0%	39.6%	13.2%	1.9%	11.3%	100.0%
		% within contact made with water	13.1%	6.1%	13.7%	7.7%	8.0%	8.5%
		% of Total	2.9%	3.4%	1.1%	.2%	1.0%	8.5%
Total		Count	137	344	51	13	75	620
		Expected Count	137.0	344.0	51.0	13.0	75.0	620.0
		% within intermidiate host	22.1%	55.5%	8.2%	2.1%	12.1%	100.0%
		% within contact made with water	100.0%	100.0%	100.0%	100.0%	100.0%	100.0%
		% of Total	22.1%	55.5%	8.2%	2.1%	12.1%	100.0%

intermidiate host * contact made with water

Crosstab

			mother complete primary education		
			yes	no	Total
intermidiate host	housefly and cocroaches	Count	189	93	282
		Expected Count	196.5	85.5	282.0
		% within intermidiate host	67.0%	33.0%	100.0%
		% within mother complete primary education	43.8%	49.5%	45.5%
		% of Total	30.5%	15.0%	45.5%
	water snail	Count	89	10	99
		Expected Count	69.0	30.0	99.0
		% within intermidiate host	89.9%	10.1%	100.0%
		% within mother complete primary education	20.6%	5.3%	16.0%
		% of Total	14.4%	1.6%	16.0%
	fish	Count	72	63	135
		Expected Count	94.1	40.9	135.0
		% within intermidiate host	53.3%	46.7%	100.0%
		% within mother complete primary education	16.7%	33.5%	21.8%
		% of Total	11.6%	10.2%	21.8%
	houserat	Count	39	12	51
		Expected Count	35.5	15.5	51.0
		% within intermidiate host	76.5%	23.5%	100.0%
		% within mother complete primary education	9.0%	6.4%	8.2%
		% of Total	6.3%	1.9%	8.2%
	i don't know	Count	43	10	53
		Expected Count	36.9	16.1	53.0

	% within intermidiate host	81.1%	18.9%	100.0%
	% within mother complete primary education	10.0%	5.3%	8.5%
	% of Total	6.9%	1.6%	8.5%
Total	Count	432	188	620
	Expected Count	432.0	188.0	620.0
	% within intermidiate host	69.7%	30.3%	100.0%
	% within mother complete primary education	100.0%	100.0%	100.0%
	% of Total	69.7%	30.3%	100.0%

Chi-Square Tests

	Value	df	Asymp. Sig. (2-sided)
Pearson Chi-Square	26.384[a]	16	.049
Likelihood Ratio	25.368	16	.064
Linear-by-Linear Association	.369	1	.543
N of Valid Cases	620		

a. 6 cells (24.0%) have expected count less than 5. The minimum expected count is 1.07.

intermidiate host * mother complete primary education

Chi-Square Tests

	Value	df	Asymp. Sig. (2-sided)
Pearson Chi-Square	41.576[a]	4	.000
Likelihood Ratio	44.898	4	.000
Linear-by-Linear Association	.490	1	.484
N of Valid Cases	620		

a. 0 cells (.0%) have expected count less than 5. The minimum expected count is 15.46.

intermidiate host * father complete primary education

Crosstab

			father complete primary education		Total
			yes	no	
intermidiate host	housefly and cocroaches	Count	214	68	282
		Expected Count	217.0	65.0	282.0
		% within intermidiate host	75.9%	24.1%	100.0%
		% within father complete primary education	44.9%	47.6%	45.5%
		% of Total	34.5%	11.0%	45.5%
	water snail	Count	92	7	99
		Expected Count	76.2	22.8	99.0
		% within intermidiate host	92.9%	7.1%	100.0%
		% within father complete primary education	19.3%	4.9%	16.0%
		% of Total	14.8%	1.1%	16.0%
	fish	Count	82	53	135
		Expected Count	103.9	31.1	135.0
		% within intermidiate host	60.7%	39.3%	100.0%
		% within father complete primary education	17.2%	37.1%	21.8%
		% of Total	13.2%	8.5%	21.8%
	houserat	Count	43	8	51
		Expected Count	39.2	11.8	51.0
		% within intermidiate host	84.3%	15.7%	100.0%
		% within father complete primary education	9.0%	5.6%	8.2%

		% of Total	6.9%	1.3%	8.2%
	i don't know	Count	46	7	53
		Expected Count	40.8	12.2	53.0
		% within intermidiate host	86.8%	13.2%	100.0%
		% within father complete primary education	9.6%	4.9%	8.5%
		% of Total	7.4%	1.1%	8.5%
Total		Count	477	143	620
		Expected Count	477.0	143.0	620.0
		% within intermidiate host	76.9%	23.1%	100.0%
		% within father complete primary education	100.0%	100.0%	100.0%
		% of Total	76.9%	23.1%	100.0%

Chi-Square Tests

	Value	df	Asymp. Sig. (2-sided)
Pearson Chi-Square	38.866[a]	4	.000
Likelihood Ratio	40.985	4	.000
Linear-by-Linear Association	.096	1	.756
N of Valid Cases	620		

a. 0 cells (.0%) have expected count less than 5. The minimum expected count is 11.76.

Crosstab

			mother's occupation				Total
			trading	farming	salary earner	unemployed	
intermidiate host	housefly and cocroaches	Count	120	73	55	34	282
		Expected Count	132.4	62.8	58.7	28.2	282.0
		% within intermidiate host	42.6%	25.9%	19.5%	12.1%	100.0%
		% within mother's occupation	41.2%	52.9%	42.6%	54.8%	45.5%
		% of Total	19.4%	11.8%	8.9%	5.5%	45.5%
	water snail	Count	67	7	18	7	99
		Expected Count	46.5	22.0	20.6	9.9	99.0
		% within intermidiate host	67.7%	7.1%	18.2%	7.1%	100.0%
		% within mother's occupation	23.0%	5.1%	14.0%	11.3%	16.0%
		% of Total	10.8%	1.1%	2.9%	1.1%	16.0%
	fish	Count	57	47	19	12	135
		Expected Count	63.4	30.0	28.1	13.5	135.0
		% within intermidiate host	42.2%	34.8%	14.1%	8.9%	100.0%
		% within mother's occupation	19.6%	34.1%	14.7%	19.4%	21.8%
		% of Total	9.2%	7.6%	3.1%	1.9%	21.8%
	houserat	Count	22	5	20	4	51
		Expected Count	23.9	11.4	10.6	5.1	51.0
		% within intermidiate host	43.1%	9.8%	39.2%	7.8%	100.0%
		% within mother's occupation	7.6%	3.6%	15.5%	6.5%	8.2%
		% of Total	3.5%	.8%	3.2%	.6%	8.2%

i don't know	Count	25	6	17	5	53
	Expected Count	24.9	11.8	11.0	5.3	53.0
	% within intermidiate host	47.2%	11.3%	32.1%	9.4%	100.0%
	% within mother's occupation	8.6%	4.3%	13.2%	8.1%	8.5%
	% of Total	4.0%	1.0%	2.7%	.8%	8.5%
Total	Count	291	138	129	62	620
	Expected Count	291.0	138.0	129.0	62.0	620.0
	% within intermidiate host	46.9%	22.3%	20.8%	10.0%	100.0%
	% within mother's occupation	100.0%	100.0%	100.0%	100.0%	100.0%
	% of Total	46.9%	22.3%	20.8%	10.0%	100.0%

intermidiate host * mother's occupation

Crosstab

			father's occupation					
			fishing	farming	trading	salary earner	wage earner	Total
intermidiate host	housefly and cocroaches	Count	11	127	75	49	20	282
		Expected Count	6.8	119.6	73.2	60.0	22.3	282.0
		% within intermidiate host	3.9%	45.0%	26.6%	17.4%	7.1%	100.0%
		% within father's occupation	73.3%	48.3%	46.6%	37.1%	40.8%	45.5%
		% of Total	1.8%	20.5%	12.1%	7.9%	3.2%	45.5%
	water snail	Count	1	38	30	21	9	99
		Expected Count	2.4	42.0	25.7	21.1	7.8	99.0
		% within intermidiate host	1.0%	38.4%	30.3%	21.2%	9.1%	100.0%
		% within father's occupation	6.7%	14.4%	18.6%	15.9%	18.4%	16.0%
		% of Total	.2%	6.1%	4.8%	3.4%	1.5%	16.0%
	fish	Count	3	69	28	25	10	135
		Expected Count	3.3	57.3	35.1	28.7	10.7	135.0
		% within intermidiate host	2.2%	51.1%	20.7%	18.5%	7.4%	100.0%
		% within father's occupation	20.0%	26.2%	17.4%	18.9%	20.4%	21.8%
		% of Total	.5%	11.1%	4.5%	4.0%	1.6%	21.8%
	houserat	Count	0	11	18	20	2	51
		Expected Count	1.2	21.6	13.2	10.9	4.0	51.0
		% within intermidiate host	.0%	21.6%	35.3%	39.2%	3.9%	100.0%
		% within father's occupation	.0%	4.2%	11.2%	15.2%	4.1%	8.2%

		% of Total	.0%	1.8%	2.9%	3.2%	.3%	8.2%
	i don't know	Count	0	18	10	17	8	53
		Expected Count	1.3	22.5	13.8	11.3	4.2	53.0
		% within intermidiate host	.0%	34.0%	18.9%	32.1%	15.1%	100.0%
		% within father's occupation	.0%	6.8%	6.2%	12.9%	16.3%	8.5%
		% of Total	.0%	2.9%	1.6%	2.7%	1.3%	8.5%
Total		Count	15	263	161	132	49	620
		Expected Count	15.0	263.0	161.0	132.0	49.0	620.0
		% within intermidiate host	2.4%	42.4%	26.0%	21.3%	7.9%	100.0%
		% within father's occupation	100.0%	100.0%	100.0%	100.0%	100.0%	100.0%
		% of Total	2.4%	42.4%	26.0%	21.3%	7.9%	100.0%

Chi-Square Tests

	Value	df	Asymp. Sig. (2-sided)
Pearson Chi-Square	56.421[a]	12	.000
Likelihood Ratio	57.478	12	.000
Linear-by-Linear Association	.055	1	.814
N of Valid Cases	620		

a. 0 cells (.0%) have expected count less than 5. The minimum expected count is 5.10.

intermidiate host * father's occupation

Chi-Square Tests

	Value	df	Asymp. Sig. (2-sided)
Pearson Chi-Square	38.239[a]	16	.001
Likelihood Ratio	39.385	16	.001
Linear-by-Linear Association	11.301	1	.001
N of Valid Cases	620		

a. 6 cells (24.0%) have expected count less than 5. The minimum expected count is 1.23.

intermidiate host * sex

Crosstab

			sex		Total
			male	female	
intermidiate host	housefly and cocroaches	Count	177	105	282
		Expected Count	164.7	117.3	282.0
		% within intermidiate host	62.8%	37.2%	100.0%
		% within sex	48.9%	40.7%	45.5%
		% of Total	28.5%	16.9%	45.5%
	water snail	Count	55	44	99
		Expected Count	57.8	41.2	99.0
		% within intermidiate host	55.6%	44.4%	100.0%
		% within sex	15.2%	17.1%	16.0%
		% of Total	8.9%	7.1%	16.0%
	fish	Count	74	61	135
		Expected Count	78.8	56.2	135.0
		% within intermidiate host	54.8%	45.2%	100.0%
		% within sex	20.4%	23.6%	21.8%
		% of Total	11.9%	9.8%	21.8%
	houserat	Count	27	24	51
		Expected Count	29.8	21.2	51.0
		% within intermidiate host	52.9%	47.1%	100.0%
		% within sex	7.5%	9.3%	8.2%
		% of Total	4.4%	3.9%	8.2%
	i don't know	Count	29	24	53

	Expected Count	30.9	22.1	53.0
	% within intermidiate host	54.7%	45.3%	100.0%
	% within sex	8.0%	9.3%	8.5%
	% of Total	4.7%	3.9%	8.5%
Total	Count	362	258	620
	Expected Count	362.0	258.0	620.0
	% within intermidiate host	58.4%	41.6%	100.0%
	% within sex	100.0%	100.0%	100.0%
	% of Total	58.4%	41.6%	100.0%

Chi-Square Tests

	Value	df	Asymp. Sig. (2-sided)
Pearson Chi-Square	4.178[a]	4	.383
Likelihood Ratio	4.188	4	.381
Linear-by-Linear Association	3.112	1	.078
N of Valid Cases	620		

a. 0 cells (.0%) have expected count less than 5. The minimum expected count is 21.22.

Crosstab

			Age distribution					Total
			<_4	5 - 9	10 - 14	15 - 19	>_20	
intermidiate host	housefly and cocroaches	Count	7	75	117	76	7	282
		Expected Count	7.7	71.0	122.8	76.4	4.1	282.0
		% within intermidiate host	2.5%	26.6%	41.5%	27.0%	2.5%	100.0%
		% within Age distribution	41.2%	48.1%	43.3%	45.2%	77.8%	45.5%
		% of Total	1.1%	12.1%	18.9%	12.3%	1.1%	45.5%
	water snail	Count	7	9	47	36	0	99
		Expected Count	2.7	24.9	43.1	26.8	1.4	99.0
		% within intermidiate host	7.1%	9.1%	47.5%	36.4%	.0%	100.0%
		% within Age distribution	41.2%	5.8%	17.4%	21.4%	.0%	16.0%
		% of Total	1.1%	1.5%	7.6%	5.8%	.0%	16.0%
	fish	Count	1	34	68	31	1	135
		Expected Count	3.7	34.0	58.8	36.6	2.0	135.0
		% within intermidiate host	.7%	25.2%	50.4%	23.0%	.7%	100.0%
		% within Age distribution	5.9%	21.8%	25.2%	18.5%	11.1%	21.8%
		% of Total	.2%	5.5%	11.0%	5.0%	.2%	21.8%
	houserat	Count	1	17	21	11	1	51
		Expected Count	1.4	12.8	22.2	13.8	.7	51.0

		% within intermidiate host	2.0%	33.3%	41.2%	21.6%	2.0%	100.0%
		% within Age distribution	5.9%	10.9%	7.8%	6.5%	11.1%	8.2%
		% of Total	.2%	2.7%	3.4%	1.8%	.2%	8.2%
	i don't know	Count	1	21	17	14	0	53
		Expected Count	1.5	13.3	23.1	14.4	.8	53.0
		% within intermidiate host	1.9%	39.6%	32.1%	26.4%	.0%	100.0%
		% within Age distribution	5.9%	13.5%	6.3%	8.3%	.0%	8.5%
		% of Total	.2%	3.4%	2.7%	2.3%	.0%	8.5%
Total		Count	17	156	270	168	9	620
		Expected Count	17.0	156.0	270.0	168.0	9.0	620.0
		% within intermidiate host	2.7%	25.2%	43.5%	27.1%	1.5%	100.0%
		% within Age distribution	100.0%	100.0%	100.0%	100.0%	100.0%	100.0%
		% of Total	2.7%	25.2%	43.5%	27.1%	1.5%	100.0%

intermidiate host * Age distribution

Chi-Square Tests

	Value	df	Asymp. Sig. (2-sided)
Pearson Chi-Square	38.354[a]	16	.001
Likelihood Ratio	41.475	16	.000
Linear-by-Linear Association	2.399	1	.121
N of Valid Cases	620		

a. 9 cells (36.0%) have expected count less than 5. The minimum expected count is .74.

Crosstab

			main source of water supply				Total
			tap	well	river	others	
source of infection	water	Count	18	64	37	1	120
		Expected Count	13.7	59.8	45.9	.6	120.0
		% within source of infection	15.0%	53.3%	30.8%	.8%	100.0%
		% within main source of water supply	25.4%	20.7%	15.6%	33.3%	19.4%
		% of Total	2.9%	10.3%	6.0%	.2%	19.4%
	playing with infected friends	Count	8	30	24	0	62
		Expected Count	7.1	30.9	23.7	.3	62.0
		% within source of infection	12.9%	48.4%	38.7%	.0%	100.0%
		% within main source of water supply	11.3%	9.7%	10.1%	.0%	10.0%
		% of Total	1.3%	4.8%	3.9%	.0%	10.0%
	food	Count	10	26	16	1	53
		Expected Count	6.1	26.4	20.3	.3	53.0
		% within source of infection	18.9%	49.1%	30.2%	1.9%	100.0%
		% within main source of	14.1%	8.4%	6.8%	33.3%	8.5%

		water supply					
		% of Total	1.6%	4.2%	2.6%	.2%	8.5%
	i don't know	Count	35	189	160	1	385
		Expected Count	44.1	191.9	147.2	1.9	385.0
		% within source of infection	9.1%	49.1%	41.6%	.3%	100.0%
		% within main source of water supply	49.3%	61.2%	67.5%	33.3%	62.1%
		% of Total	5.6%	30.5%	25.8%	.2%	62.1%
Total		Count	71	309	237	3	620
		Expected Count	71.0	309.0	237.0	3.0	620.0
		% within source of infection	11.5%	49.8%	38.2%	.5%	100.0%
		% within main source of water supply	100.0%	100.0%	100.0%	100.0%	100.0%
		% of Total	11.5%	49.8%	38.2%	.5%	100.0%

source of infection * main source of water supply

Crosstab

			contact made with water					Total
			play or bath	washing	agricultural work	fishing	no contact	
source of infection	water	Count	29	64	7	2	18	120
		Expected Count	26.5	66.6	9.9	2.5	14.5	120.0
		% within source of infection	24.2%	53.3%	5.8%	1.7%	15.0%	100.0%
		% within contact made with water	21.2%	18.6%	13.7%	15.4%	24.0%	19.4%
		% of Total	4.7%	10.3%	1.1%	.3%	2.9%	19.4%
	playing with infected friends	Count	13	38	2	5	4	62
		Expected Count	13.7	34.4	5.1	1.3	7.5	62.0
		% within source of infection	21.0%	61.3%	3.2%	8.1%	6.5%	100.0%
		% within contact made with water	9.5%	11.0%	3.9%	38.5%	5.3%	10.0%
		% of Total	2.1%	6.1%	.3%	.8%	.6%	10.0%
	food	Count	16	26	1	4	6	53
		Expected Count	11.7	29.4	4.4	1.1	6.4	53.0
		% within source of infection	30.2%	49.1%	1.9%	7.5%	11.3%	100.0%
		% within contact made with water	11.7%	7.6%	2.0%	30.8%	8.0%	8.5%
		% of Total	2.6%	4.2%	.2%	.6%	1.0%	8.5%
		Count	79	216	41	2	47	385

	i don't know	Expected Count	85.1	213.6	31.7	8.1	46.6	385.0
		% within source of infection	20.5%	56.1%	10.6%	.5%	12.2%	100.0%
		% within contact made with water	57.7%	62.8%	80.4%	15.4%	62.7%	62.1%
		% of Total	12.7%	34.8%	6.6%	.3%	7.6%	62.1%
Total		Count	137	344	51	13	75	620
		Expected Count	137.0	344.0	51.0	13.0	75.0	620.0
		% within source of infection	22.1%	55.5%	8.2%	2.1%	12.1%	100.0%
		% within contact made with water	100.0%	100.0%	100.0%	100.0%	100.0%	100.0%
		% of Total	22.1%	55.5%	8.2%	2.1%	12.1%	100.0%

Chi-Square Tests

	Value	df	Asymp. Sig. (2-sided)
Pearson Chi-Square	13.115[a]	9	.157
Likelihood Ratio	12.277	9	.198
Linear-by-Linear Association	5.246	1	.022
N of Valid Cases	620		

a. 4 cells (25.0%) have expected count less than 5. The minimum expected count is .26.

source of infection * contact made with water

Crosstab

			mother complete primary education		
			yes	no	Total
source of infection	water	Count	98	22	120
		Expected Count	83.6	36.4	120.0
		% within source of infection	81.7%	18.3%	100.0%
		% within mother complete primary education	22.7%	11.7%	19.4%
		% of Total	15.8%	3.5%	19.4%
	playing with infected friends	Count	40	22	62
		Expected Count	43.2	18.8	62.0
		% within source of infection	64.5%	35.5%	100.0%
		% within mother complete primary education	9.3%	11.7%	10.0%
		% of Total	6.5%	3.5%	10.0%
	food	Count	43	10	53
		Expected Count	36.9	16.1	53.0
		% within source of infection	81.1%	18.9%	100.0%
		% within mother complete primary education	10.0%	5.3%	8.5%
		% of Total	6.9%	1.6%	8.5%
	i don't know	Count	251	134	385
		Expected Count	268.3	116.7	385.0
		% within source of infection	65.2%	34.8%	100.0%

	% within mother complete primary education	58.1%	71.3%	62.1%
	% of Total	40.5%	21.6%	62.1%
Total	Count	432	188	620
	Expected Count	432.0	188.0	620.0
	% within source of infection	69.7%	30.3%	100.0%
	% within mother complete primary education	100.0%	100.0%	100.0%
	% of Total	69.7%	30.3%	100.0%

Chi-Square Tests

	Value	df	Asymp. Sig. (2-sided)
Pearson Chi-Square	36.441[a]	12	.000
Likelihood Ratio	32.686	12	.001
Linear-by-Linear Association	.003	1	.958
N of Valid Cases	620		

a. 4 cells (20.0%) have expected count less than 5. The minimum expected count is 1.11.

source of infection * mother complete primary education

Chi-Square Tests

	Value	df	Asymp. Sig. (2-sided)
Pearson Chi-Square	15.899[a]	3	.001
Likelihood Ratio	16.908	3	.001
Linear-by-Linear Association	9.465	1	.002
N of Valid Cases	620		

a. 0 cells (.0%) have expected count less than 5. The minimum expected count is 16.07.

source of infection * father complete primary education

Crosstab

			father complete primary education		Total
			yes	no	
source of infection	water	Count	104	16	120
		Expected Count	92.3	27.7	120.0
		% within source of infection	86.7%	13.3%	100.0%
		% within father complete primary education	21.8%	11.2%	19.4%
		% of Total	16.8%	2.6%	19.4%
	playing with infected friends	Count	51	11	62
		Expected Count	47.7	14.3	62.0
		% within source of infection	82.3%	17.7%	100.0%
		% within father complete primary education	10.7%	7.7%	10.0%
		% of Total	8.2%	1.8%	10.0%
	food	Count	46	7	53
		Expected Count	40.8	12.2	53.0
		% within source of infection	86.8%	13.2%	100.0%
		% within father complete primary education	9.6%	4.9%	8.5%
		% of Total	7.4%	1.1%	8.5%
	i don't know	Count	276	109	385
		Expected Count	296.2	88.8	385.0

	% within source of infection	71.7%	28.3%	100.0%
	% within father complete primary education	57.9%	76.2%	62.1%
	% of Total	44.5%	17.6%	62.1%
Total	Count	477	143	620
	Expected Count	477.0	143.0	620.0
	% within source of infection	76.9%	23.1%	100.0%
	% within father complete primary education	100.0%	100.0%	100.0%
	% of Total	76.9%	23.1%	100.0%

Chi-Square Tests

	Value	df	Asymp. Sig. (2-sided)
Pearson Chi-Square	16.269[a]	3	.001
Likelihood Ratio	17.266	3	.001
Linear-by-Linear Association	13.487	1	.000
N of Valid Cases	620		

a. 0 cells (.0%) have expected count less than 5. The minimum expected count is 12.22.

source of infection * mother's occupation

Crosstab

			mother's occupation				Total
			trading	farming	salary earner	unemployed	
source of infection	water	Count	65	18	26	11	120
		Expected Count	56.3	26.7	25.0	12.0	120.0
		% within source of infection	54.2%	15.0%	21.7%	9.2%	100.0%
		% within mother's occupation	22.3%	13.0%	20.2%	17.7%	19.4%
		% of Total	10.5%	2.9%	4.2%	1.8%	19.4%
	playing with infected friends	Count	27	13	18	4	62
		Expected Count	29.1	13.8	12.9	6.2	62.0
		% within source of infection	43.5%	21.0%	29.0%	6.5%	100.0%
		% within mother's occupation	9.3%	9.4%	14.0%	6.5%	10.0%
		% of Total	4.4%	2.1%	2.9%	.6%	10.0%
	food	Count	28	5	13	7	53
		Expected Count	24.9	11.8	11.0	5.3	53.0
		% within source of infection	52.8%	9.4%	24.5%	13.2%	100.0%
		% within mother's occupation	9.6%	3.6%	10.1%	11.3%	8.5%
		% of Total	4.5%	.8%	2.1%	1.1%	8.5%
	i don't know	Count	171	102	72	40	385

	Expected Count	180.7	85.7	80.1	38.5	385.0
	% within source of infection	44.4%	26.5%	18.7%	10.4%	100.0%
	% within mother's occupation	58.8%	73.9%	55.8%	64.5%	62.1%
	% of Total	27.6%	16.5%	11.6%	6.5%	62.1%
Total	Count	291	138	129	62	620
	Expected Count	291.0	138.0	129.0	62.0	620.0
	% within source of infection	46.9%	22.3%	20.8%	10.0%	100.0%
	% within mother's occupation	100.0%	100.0%	100.0 %	100.0%	100.0%
	% of Total	46.9%	22.3%	20.8%	10.0%	100.0%

Crosstab

			father's occupation					Total
			fishing	farming	trading	salary earner	wage earner	
source of infection	water	Count	2	48	34	29	7	120
		Expected Count	2.9	50.9	31.2	25.5	9.5	120.0
		% within source of infection	1.7%	40.0%	28.3%	24.2%	5.8%	100.0%
		% within father's occupation	13.3%	18.3%	21.1%	22.0%	14.3%	19.4%
		% of Total	.3%	7.7%	5.5%	4.7%	1.1%	19.4%
	playing with infected friends	Count	7	21	17	11	6	62
		Expected Count	1.5	26.3	16.1	13.2	4.9	62.0
		% within source of infection	11.3%	33.9%	27.4%	17.7%	9.7%	100.0%
		% within father's occupation	46.7%	8.0%	10.6%	8.3%	12.2%	10.0%
		% of Total	1.1%	3.4%	2.7%	1.8%	1.0%	10.0%
	food	Count	1	16	16	15	5	53
		Expected Count	1.3	22.5	13.8	11.3	4.2	53.0
		% within source of infection	1.9%	30.2%	30.2%	28.3%	9.4%	100.0%
		% within father's occupation	6.7%	6.1%	9.9%	11.4%	10.2%	8.5%
		% of Total	.2%	2.6%	2.6%	2.4%	.8%	8.5%
		Count	5	178	94	77	31	385

	i don't know	Expected Count	9.3	163.3	100.0	82.0	30.4	385.0
		% within source of infection	1.3%	46.2%	24.4%	20.0%	8.1%	100.0%
		% within father's occupation	33.3%	67.7%	58.4%	58.3%	63.3%	62.1%
		% of Total	.8%	28.7%	15.2%	12.4%	5.0%	62.1%
Total		Count	15	263	161	132	49	620
		Expected Count	15.0	263.0	161.0	132.0	49.0	620.0
		% within source of infection	2.4%	42.4%	26.0%	21.3%	7.9%	100.0%
		% within father's occupation	100.0%	100.0%	100.0%	100.0%	100.0%	100.0%
		% of Total	2.4%	42.4%	26.0%	21.3%	7.9%	100.0%

Chi-Square Tests

	Value	df	Asymp. Sig. (2-sided)
Pearson Chi-Square	17.007[a]	9	.049
Likelihood Ratio	18.078	9	.034
Linear-by-Linear Association	.451	1	.502
N of Valid Cases	620		

a. 0 cells (.0%) have expected count less than 5. The minimum expected count is 5.30.

source of infection * father's occupation

Crosstab

			sex		Total
			male	female	
source of infection	water	Count	72	48	120
		Expected Count	70.1	49.9	120.0
		% within source of infection	60.0%	40.0%	100.0%
		% within sex	19.9%	18.6%	19.4%
		% of Total	11.6%	7.7%	19.4%
	playing with infected friends	Count	39	23	62
		Expected Count	36.2	25.8	62.0
		% within source of infection	62.9%	37.1%	100.0%
		% within sex	10.8%	8.9%	10.0%
		% of Total	6.3%	3.7%	10.0%
	food	Count	31	22	53
		Expected Count	30.9	22.1	53.0
		% within source of infection	58.5%	41.5%	100.0%
		% within sex	8.6%	8.5%	8.5%
		% of Total	5.0%	3.5%	8.5%
	i don't know	Count	220	165	385
		Expected Count	224.8	160.2	385.0
		% within source of infection	57.1%	42.9%	100.0%
		% within sex	60.8%	64.0%	62.1%
		% of Total	35.5%	26.6%	62.1%
Total		Count	362	258	620
		Expected Count	362.0	258.0	620.0

	% within source of infection	58.4%	41.6%	100.0%
	% within sex	100.0%	100.0%	100.0%
	% of Total	58.4%	41.6%	100.0%

Chi-Square Tests

	Value	df	Asymp. Sig. (2-sided)
Pearson Chi-Square	31.385[a]	12	.002
Likelihood Ratio	22.403	12	.033
Linear-by-Linear Association	.117	1	.732
N of Valid Cases	620		

a. 5 cells (25.0%) have expected count less than 5. The minimum expected count is 1.28.

source of infection * sex

Chi-Square Tests

	Value	df	Asymp. Sig. (2-sided)
Pearson Chi-Square	.894[a]	3	.827
Likelihood Ratio	.901	3	.825
Linear-by-Linear Association	.589	1	.443
N of Valid Cases	620		

Chi-Square Tests

	Value	df	Asymp. Sig. (2-sided)
Pearson Chi-Square	.894[a]	3	.827
Likelihood Ratio	.901	3	.825
Linear-by-Linear Association	.589	1	.443

a. 0 cells (.0%) have expected count less than 5. The minimum expected count is 22.05.

source of infection * Age distribution

Crosstab

			Age distribution					Total
			<_4	5 - 9	10 - 14	15 - 19	>_20	
source of infection	water	Count	2	24	59	33	2	120
		Expected Count	3.3	30.2	52.3	32.5	1.7	120.0
		% within source of infection	1.7%	20.0%	49.2%	27.5%	1.7%	100.0%
		% within Age distribution	11.8%	15.4%	21.9%	19.6%	22.2%	19.4%
		% of Total	.3%	3.9%	9.5%	5.3%	.3%	19.4%
	playing with infected friends	Count	1	13	27	20	1	62
		Expected Count	1.7	15.6	27.0	16.8	.9	62.0
		% within source of infection	1.6%	21.0%	43.5%	32.3%	1.6%	100.0%
		% within Age distribution	5.9%	8.3%	10.0%	11.9%	11.1%	10.0%
		% of Total	.2%	2.1%	4.4%	3.2%	.2%	10.0%
	food	Count	0	13	29	11	0	53
		Expected Count	1.5	13.3	23.1	14.4	.8	53.0
		% within source of infection	.0%	24.5%	54.7%	20.8%	.0%	100.0%
		% within Age distribution	.0%	8.3%	10.7%	6.5%	.0%	8.5%
		% of Total	.0%	2.1%	4.7%	1.8%	.0%	8.5%
	i don't know	Count	14	106	155	104	6	385
		Expected Count	10.6	96.9	167.7	104.3	5.6	385.0
		% within source of infection	3.6%	27.5%	40.3%	27.0%	1.6%	100.0%

	% within Age distribution	82.4%	67.9%	57.4%	61.9%	66.7%	62.1%
	% of Total	2.3%	17.1%	25.0%	16.8%	1.0%	62.1%
Total	Count	17	156	270	168	9	620
	Expected Count	17.0	156.0	270.0	168.0	9.0	620.0
	% within source of infection	2.7%	25.2%	43.5%	27.1%	1.5%	100.0%
	% within Age distribution	100.0%	100.0%	100.0%	100.0%	100.0%	100.0%
	% of Total	2.7%	25.2%	43.5%	27.1%	1.5%	100.0%

Crosstab

			main source of water supply				Total
			tap	well	river	others	
route of schistosome infection	contact with contaminated natural water	Count	23	74	30	1	128
		Expected Count	14.7	63.8	48.9	.6	128.0
		% within route of schistosome infection	18.0%	57.8%	23.4%	.8%	100.0%
		% within main source of water supply	32.4%	23.9%	12.7%	33.3%	20.6%
		% of Total	3.7%	11.9%	4.8%	.2%	20.6%
	eating unhygienic food	Count	9	17	14	0	40
		Expected Count	4.6	19.9	15.3	.2	40.0
		% within route of schistosome infection	22.5%	42.5%	35.0%	.0%	100.0%
		% within main source of water supply	12.7%	5.5%	5.9%	.0%	6.5%
		% of Total	1.5%	2.7%	2.3%	.0%	6.5%
	playing with soil	Count	5	20	20	0	45
		Expected Count	5.2	22.4	17.2	.2	45.0
		% within route of schistosome infection	11.1%	44.4%	44.4%	.0%	100.0%
		% within main source of water supply	7.0%	6.5%	8.4%	.0%	7.3%
		% of Total	.8%	3.2%	3.2%	.0%	7.3%
	i don't know	Count	34	198	173	2	407

		Expected Count	46.6	202.8	155.6	2.0	407.0
		% within route of schistosome infection	8.4%	48.6%	42.5%	.5%	100.0%
		% within main source of water supply	47.9%	64.1%	73.0%	66.7%	65.6%
		% of Total	5.5%	31.9%	27.9%	.3%	65.6%
Total		Count	71	309	237	3	620
		Expected Count	71.0	309.0	237.0	3.0	620.0
		% within route of schistosome infection	11.5%	49.8%	38.2%	.5%	100.0%
		% within main source of water supply	100.0%	100.0%	100.0%	100.0%	100.0%
		% of Total	11.5%	49.8%	38.2%	.5%	100.0%

Chi-Square Tests

	Value	df	Asymp. Sig. (2-sided)
Pearson Chi-Square	11.541[a]	12	.483
Likelihood Ratio	13.795	12	.314
Linear-by-Linear Association	2.848	1	.092
N of Valid Cases	620		

a. 6 cells (30.0%) have expected count less than 5. The minimum expected count is .77.

route of schistosome infection * main source of water supply

Chi-Square Tests

	Value	df	Asymp. Sig. (2-sided)
Pearson Chi-Square	25.354[a]	9	.003
Likelihood Ratio	25.481	9	.002
Linear-by-Linear Association	19.264	1	.000
N of Valid Cases	620		

a. 5 cells (31.3%) have expected count less than 5. The minimum expected count is .19.

route of schistosome infection * contact made with water

Crosstab

			contact made with water					Total
			play or bath	washing	agricultural work	fishing	no contact	
route of schistosome infection	contact with contaminated natural water	Count	30	58	10	1	29	128
		Expected Count	28.3	71.0	10.5	2.7	15.5	128.0
		% within route of schistosome infection	23.4%	45.3%	7.8%	.8%	22.7%	100.0%
		% within contact made with water	21.9%	16.9%	19.6%	7.7%	38.7%	20.6%
		% of Total	4.8%	9.4%	1.6%	.2%	4.7%	20.6%
	eating unhygienic food	Count	11	22	1	3	3	40
		Expected Count	8.8	22.2	3.3	.8	4.8	40.0
		% within route of schistosome infection	27.5%	55.0%	2.5%	7.5%	7.5%	100.0%
		% within contact made with water	8.0%	6.4%	2.0%	23.1%	4.0%	6.5%
		% of Total	1.8%	3.5%	.2%	.5%	.5%	6.5%
	playing with soil	Count	9	30	3	2	1	45
		Expected Count	9.9	25.0	3.7	.9	5.4	45.0
		% within route of	20.0%	66.7%	6.7%	4.4%	2.2%	100.0%

		schistosome infection						
		% within contact made with water	6.6%	8.7%	5.9%	15.4%	1.3%	7.3%
		% of Total	1.5%	4.8%	.5%	.3%	.2%	7.3%
	i don't know	Count	87	234	37	7	42	407
		Expected Count	89.9	225.8	33.5	8.5	49.2	407.0
		% within route of schistosome infection	21.4%	57.5%	9.1%	1.7%	10.3%	100.0%
		% within contact made with water	63.5%	68.0%	72.5%	53.8%	56.0%	65.6%
		% of Total	14.0%	37.7%	6.0%	1.1%	6.8%	65.6%
Total		Count	137	344	51	13	75	620
		Expected Count	137.0	344.0	51.0	13.0	75.0	620.0
		% within route of schistosome infection	22.1%	55.5%	8.2%	2.1%	12.1%	100.0%
		% within contact made with water	100.0%	100.0%	100.0%	100.0%	100.0%	100.0%
		% of Total	22.1%	55.5%	8.2%	2.1%	12.1%	100.0%

Chi-Square Tests

	Value	df	Asymp. Sig. (2-sided)
Pearson Chi-Square	31.913[a]	12	.001
Likelihood Ratio	29.995	12	.003
Linear-by-Linear Association	5.155	1	.023
N of Valid Cases	620		

a. 6 cells (30.0%) have expected count less than 5. The minimum expected count is .84.

route of schistosome infection * mother complete primary education

Crosstab

			mother complete primary education		Total
			yes	no	
route of schistosome infection	contact with contaminated natural water	Count	102	26	128
		Expected Count	89.2	38.8	128.0
		% within route of schistosome infection	79.7%	20.3%	100.0%
		% within mother complete primary education	23.6%	13.8%	20.6%
		% of Total	16.5%	4.2%	20.6%
	eating unhygienic food	Count	31	9	40
		Expected Count	27.9	12.1	40.0
		% within route of schistosome infection	77.5%	22.5%	100.0%
		% within mother complete primary education	7.2%	4.8%	6.5%
		% of Total	5.0%	1.5%	6.5%
	playing with soil	Count	32	13	45
		Expected Count	31.4	13.6	45.0
		% within route of schistosome infection	71.1%	28.9%	100.0%
		% within mother complete primary education	7.4%	6.9%	7.3%
		% of Total	5.2%	2.1%	7.3%
	i don't know	Count	267	140	407

	Expected Count	283.6	123.4	407.0
	% within route of schistosome infection	65.6%	34.4%	100.0%
	% within mother complete primary education	61.8%	74.5%	65.6%
	% of Total	43.1%	22.6%	65.6%
Total	Count	432	188	620
	Expected Count	432.0	188.0	620.0
	% within route of schistosome infection	69.7%	30.3%	100.0%
	% within mother complete primary education	100.0%	100.0%	100.0%
	% of Total	69.7%	30.3%	100.0%

Chi-Square Tests

	Value	df	Asymp. Sig. (2-sided)
Pearson Chi-Square	10.472[a]	3	.015
Likelihood Ratio	10.946	3	.012
Linear-by-Linear Association	10.345	1	.001
N of Valid Cases	620		

a. 0 cells (.0%) have expected count less than 5. The minimum expected count is 12.13.

Crosstab

			father complete primary education		Total
			yes	no	
route of schistosome infection	contact with contaminated natural water	Count	110	18	128
		Expected Count	98.5	29.5	128.0
		% within route of schistosome infection	85.9%	14.1%	100.0%
		% within father complete primary education	23.1%	12.6%	20.6%
		% of Total	17.7%	2.9%	20.6%
	eating unhygienic food	Count	37	3	40
		Expected Count	30.8	9.2	40.0
		% within route of schistosome infection	92.5%	7.5%	100.0%
		% within father complete primary education	7.8%	2.1%	6.5%
		% of Total	6.0%	.5%	6.5%
	playing with soil	Count	37	8	45
		Expected Count	34.6	10.4	45.0
		% within route of schistosome infection	82.2%	17.8%	100.0%
		% within father complete primary education	7.8%	5.6%	7.3%
		% of Total	6.0%	1.3%	7.3%
	i don't know	Count	293	114	407
		Expected Count	313.1	93.9	407.0
		% within route of schistosome infection	72.0%	28.0%	100.0%

	% within father complete primary education	61.4%	79.7%	65.6%
	% of Total	47.3%	18.4%	65.6%
Total	Count	477	143	620
	Expected Count	477.0	143.0	620.0
	% within route of schistosome infection	76.9%	23.1%	100.0%
	% within father complete primary education	100.0%	100.0%	100.0%
	% of Total	76.9%	23.1%	100.0%

route of schistosome infection * father complete primary education

Crosstab

			mother's occupation				Total
			trading	farming	salary earner	unemployed	
route of schistosome infection	contact with contaminated natural water	Count	65	14	30	19	128
		Expected Count	60.1	28.5	26.6	12.8	128.0
		% within route of schistosome infection	50.8%	10.9%	23.4%	14.8%	100.0%
		% within mother's occupation	22.3%	10.1%	23.3%	30.6%	20.6%
		% of Total	10.5%	2.3%	4.8%	3.1%	20.6%
	eating unhygienic food	Count	23	5	11	1	40
		Expected Count	18.8	8.9	8.3	4.0	40.0
		% within route of schistosome infection	57.5%	12.5%	27.5%	2.5%	100.0%
		% within mother's occupation	7.9%	3.6%	8.5%	1.6%	6.5%
		% of Total	3.7%	.8%	1.8%	.2%	6.5%
	playing with soil	Count	24	10	7	4	45
		Expected Count	21.1	10.0	9.4	4.5	45.0
		% within route of schistosome infection	53.3%	22.2%	15.6%	8.9%	100.0%
		% within mother's occupation	8.2%	7.2%	5.4%	6.5%	7.3%
		% of Total	3.9%	1.6%	1.1%	.6%	7.3%

	i don't know	Count	179	109	81	38	407
		Expected Count	191.0	90.6	84.7	40.7	407.0
		% within route of schistosome infection	44.0%	26.8%	19.9%	9.3%	100.0%
		% within mother's occupation	61.5%	79.0%	62.8%	61.3%	65.6%
		% of Total	28.9%	17.6%	13.1%	6.1%	65.6%
Total		Count	291	138	129	62	620
		Expected Count	291.0	138.0	129.0	62.0	620.0
		% within route of schistosome infection	46.9%	22.3%	20.8%	10.0%	100.0%
		% within mother's occupation	100.0%	100.0%	100.0%	100.0%	100.0%
		% of Total	46.9%	22.3%	20.8%	10.0%	100.0%

Chi-Square Tests

	Value	df	Asymp. Sig. (2-sided)
Pearson Chi-Square	17.624[a]	3	.001
Likelihood Ratio	19.536	3	.000
Linear-by-Linear Association	14.619	1	.000
N of Valid Cases	620		

a. 0 cells (.0%) have expected count less than 5. The minimum expected count is 9.23.

route of schistosome infection * mother's occupation

Crosstab

			father's occupation					
			fishing	farming	trading	salary earner	wage earner	Total
route of schistosome infection	contact with contaminated natural water	Count	1	38	34	40	15	128
		Expected Count	3.1	54.3	33.2	27.3	10.1	128.0
		% within route of schistosome infection	.8%	29.7%	26.6%	31.2%	11.7%	100.0%
		% within father's occupation	6.7%	14.4%	21.1%	30.3%	30.6%	20.6%
		% of Total	.2%	6.1%	5.5%	6.5%	2.4%	20.6%
	eating unhygienic food	Count	2	10	17	10	1	40
		Expected Count	1.0	17.0	10.4	8.5	3.2	40.0
		% within route of schistosome infection	5.0%	25.0%	42.5%	25.0%	2.5%	100.0%
		% within father's occupation	13.3%	3.8%	10.6%	7.6%	2.0%	6.5%
		% of Total	.3%	1.6%	2.7%	1.6%	.2%	6.5%
	playing with soil	Count	5	25	13	2	0	45
		Expected Count	1.1	19.1	11.7	9.6	3.6	45.0
		% within route of schistosome infection	11.1%	55.6%	28.9%	4.4%	.0%	100.0%
		% within father's occupation	33.3%	9.5%	8.1%	1.5%	.0%	7.3%
		% of Total	.8%	4.0%	2.1%	.3%	.0%	7.3%
	i don't know	Count	7	190	97	80	33	407
		Expected Count	9.8	172.6	105.7	86.7	32.2	407.0

	% within route of schistosome infection	1.7%	46.7%	23.8%	19.7%	8.1%	100.0%
	% within father's occupation	46.7%	72.2%	60.2%	60.6%	67.3%	65.6%
	% of Total	1.1%	30.6%	15.6%	12.9%	5.3%	65.6%
Total	Count	15	263	161	132	49	620
	Expected Count	15.0	263.0	161.0	132.0	49.0	620.0
	% within route of schistosome infection	2.4%	42.4%	26.0%	21.3%	7.9%	100.0%
	% within father's occupation	100.0%	100.0%	100.0%	100.0%	100.0%	100.0%
	% of Total	2.4%	42.4%	26.0%	21.3%	7.9%	100.0%

Chi-Square Tests

	Value	df	Asymp. Sig. (2-sided)
Pearson Chi-Square	22.858[a]	9	.007
Likelihood Ratio	25.148	9	.003
Linear-by-Linear Association	.124	1	.725
N of Valid Cases	620		

a. 2 cells (12.5%) have expected count less than 5. The minimum expected count is 4.00.

route of schistosome infection * father's occupation

Chi-Square Tests

	Value	df	Asymp. Sig. (2-sided)
Pearson Chi-Square	53.957[a]	12	.000
Likelihood Ratio	53.789	12	.000
Linear-by-Linear Association	11.214	1	.001
N of Valid Cases	620		

a. 5 cells (25.0%) have expected count less than 5. The minimum expected count is .97.

route of schistosome infection * sex

Crosstab

			sex		Total
			male	female	
route of schistosome infection	contact with contaminated natural water	Count	80	48	128
		Expected Count	74.7	53.3	128.0
		% within route of schistosome infection	62.5%	37.5%	100.0%
		% within sex	22.1%	18.6%	20.6%
		% of Total	12.9%	7.7%	20.6%
	eating unhygienic food	Count	22	18	40
		Expected Count	23.4	16.6	40.0
		% within route of schistosome infection	55.0%	45.0%	100.0%
		% within sex	6.1%	7.0%	6.5%
		% of Total	3.5%	2.9%	6.5%
	playing with soil	Count	21	24	45
		Expected Count	26.3	18.7	45.0
		% within route of schistosome infection	46.7%	53.3%	100.0%
		% within sex	5.8%	9.3%	7.3%
		% of Total	3.4%	3.9%	7.3%
	i don't know	Count	239	168	407
		Expected Count	237.6	169.4	407.0
		% within route of schistosome infection	58.7%	41.3%	100.0%
		% within sex	66.0%	65.1%	65.6%

	% of Total	38.5%	27.1%	65.6%
Total	Count	362	258	620
	Expected Count	362.0	258.0	620.0
	% within route of schistosome infection	58.4%	41.6%	100.0%
	% within sex	100.0%	100.0%	100.0%
	% of Total	58.4%	41.6%	100.0%

Chi-Square Tests

	Value	df	Asymp. Sig. (2-sided)
Pearson Chi-Square	3.643[a]	3	.303
Likelihood Ratio	3.608	3	.307
Linear-by-Linear Association	.267	1	.605
N of Valid Cases	620		

a. 0 cells (.0%) have expected count less than 5. The minimum expected count is 16.65.

route of schistosome infection * Age distribution

Crosstab

			Age distribution					Total
			<_4	5 - 9	10 - 14	15 - 19	>_20	
route of schistosome infection	contact with contaminated natural water	Count	8	32	59	29	0	128
		Expected Count	3.5	32.2	55.7	34.7	1.9	128.0
		% within route of schistosome infection	6.2%	25.0%	46.1%	22.7%	.0%	100.0%
		% within Age distribution	47.1%	20.5%	21.9%	17.3%	.0%	20.6%
		% of Total	1.3%	5.2%	9.5%	4.7%	.0%	20.6%
	eating unhygienic food	Count	0	6	22	11	1	40
		Expected Count	1.1	10.1	17.4	10.8	.6	40.0
		% within route of schistosome infection	.0%	15.0%	55.0%	27.5%	2.5%	100.0%
		% within Age distribution	.0%	3.8%	8.1%	6.5%	11.1%	6.5%
		% of Total	.0%	1.0%	3.5%	1.8%	.2%	6.5%
	playing with soil	Count	0	8	22	13	2	45
		Expected Count	1.2	11.3	19.6	12.2	.7	45.0
		% within route of schistosome infection	.0%	17.8%	48.9%	28.9%	4.4%	100.0%
		% within Age distribution	.0%	5.1%	8.1%	7.7%	22.2%	7.3%
		% of Total	.0%	1.3%	3.5%	2.1%	.3%	7.3%
	i don't know	Count	9	110	167	115	6	407
		Expected Count	11.2	102.4	177.2	110.3	5.9	407.0

	% within route of schistosome infection	2.2%	27.0%	41.0%	28.3%	1.5%	100.0%
	% within Age distribution	52.9%	70.5%	61.9%	68.5%	66.7%	65.6%
	% of Total	1.5%	17.7%	26.9%	18.5%	1.0%	65.6%
Total	Count	17	156	270	168	9	620
	Expected Count	17.0	156.0	270.0	168.0	9.0	620.0
	% within route of schistosome infection	2.7%	25.2%	43.5%	27.1%	1.5%	100.0%
	% within Age distribution	100.0%	100.0%	100.0%	100.0%	100.0%	100.0%
	% of Total	2.7%	25.2%	43.5%	27.1%	1.5%	100.0%

Chi-Square Tests

	Value	df	Asymp. Sig. (2-sided)
Pearson Chi-Square	20.084[a]	12	.066
Likelihood Ratio	22.045	12	.037
Linear-by-Linear Association	1.453	1	.228
N of Valid Cases	620		

a. 6 cells (30.0%) have expected count less than 5. The minimum expected count is .58.
